# Gene Expression Trajectories from Normal Nonsmokers to COPD Smokers and Disease Progression Discriminant Modeling in Response to Cigarette Smoking

**DOI:** 10.1155/2022/9354286

**Published:** 2022-09-14

**Authors:** Zili Zhang, Sifan Chen, Qiongqiong Li, Defu Li, Yuanyuan Li, Xiaohui Xie, Liang Yuan, Zeqiang Lin, Fanjie Lin, Xinguang Wei, Yaowei Fang, Jian Wang, Wenju Lu

**Affiliations:** ^1^State Key Laboratory of Respiratory Diseases, Guangdong Key Laboratory of Vascular Diseases, National Clinical Research Center for Respiratory Diseases, Guangzhou Institute of Respiratory Health, The First Affiliated Hospital of Guangzhou Medical University, Guangzhou, Guangdong, China; ^2^Guangdong Provincial Key Laboratory of Malignant Tumor Epigenetics and Gene Regulation, Medical Research Center, Sun Yat-Sen Memorial Hospital, Sun Yat-Sen University, Guangzhou, China; ^3^The Fifth Clinical School, The Fifth Affiliated Hospital of Guangzhou Medical University, Guangzhou, Guangdong, China; ^4^Department of Respiratory Medicine, The Sixth Affiliated Hospital of Guangzhou Medical University Qingyuan People's Hospital, Qingyuan, China

## Abstract

**Background:**

Cigarette smoking (CS) is considered to the predominant risk factor contributing to the etiopathogenesis of chronic obstructive pulmonary disease (COPD); meanwhile, genetic predisposition likely plays a role in determining disease susceptibility.

**Objectives:**

We aimed to investigate gene expression trajectories from normal nonsmokers to COPD smokers and disease progression discriminant modeling in response to cigarette smoking.

**Methods:**

Small airway epithelial samples of human with different smoking status using fiberoptic bronchoscopy and corresponding rat lung tissues following 0, 3, and 6 months of CS exposure were obtained. The expression of the significant overlapping genes between human and rats was confirmed in 16HBE cells, rat lung tissues, and human peripheral PBMC using qRT-PCR. Binary logistic regression analysis was carried out to establish discrimination models.

**Results:**

The integrated bioinformatic analysis of 8 human GEO datasets (293 individuals) and 9 rat transcriptome databases revealed 13 overlapping genes between humans and rats in response to smoking exposure during COPD progression. Of these, 5 genes (AKR1C3/Akr1c3, ERP27/Erp27, AHRR/Ahrr, KCNMB2/Kcnmb2, and MRC1/Mrc1) were consistently identified in both the human and rat and validated by qRT-PCR. Among them, ERP27/Erp27, KCNMB2/Kcnmb2, and MRC1/Mrc1 were newly identified. On the basis of the overlapping gene panel, discriminant models were established with the receiver operating characteristic curve (AUC) of 0.98 (AKR1C3/Akr1c3 + ERP27/Erp27) and 0.99 (AHRR/Ahrr + KCNMB2/Kcnmb2) in differentiating progressive COPD from normal nonsmokers. In addition, we also found that DEG obtained from each expression profile dataset was better than combined analysis as more genes could be identified.

**Conclusion:**

This study identified 5 DEG candidates of COPD progression in response to smoking and developed effective and convenient discriminant models that can accurately predict the disease progression.

## 1. Background

Chronic obstructive pulmonary disease (COPD) is a highly prevalent obstructive pulmonary disorder characterized by partially reversible, persistent airflow limitation associated with chronic airway inflammation, and emphysema [1, 2]. According to the World Health Organization, COPD is predicted to become the third leading cause of mortality worldwide by 2030 [3]. Unless imperative global action is taken, experts warn that the deaths from COPD are expected to rise by over 30% in the next few decades [4]. Cigarette smoking (CS) is considered to the predominant risk factor contributing to the etiopathogenesis of COPD; meanwhile, genetic predisposition likely plays a role in determining disease susceptibility [5, 6].

The rapid advancements of genome-wide association studies (GWAS), access to enormous whole-genome sequencing data, and the development of strategies to manage and analyze the genetic data have tremendously advanced our understanding of genetic factors that play a crucial role in COPD susceptibility, over the past decade. For instance, multiple collaborative studies have identified more than 20 genetic loci associated with the risk of COPD [6]. These regions of the genome may harbor functional genes related to COPD susceptibility and severity. However, thus far, GWAS results have been shown to be less consistent as most of the findings fail to meet genome-wide significance or replicate; besides, findings below genome-wide statistical significance remain uncertain importance.

The Gene Expression Omnibus (GEO) repository at the National Center of Biotechnology Information (NCBI) (GEO, http://www.ncbi.nlm.nih.gov/geo/) is an international public functional data repository for submission, storage, and integration of high-throughput microarray/gene expression, next-generation sequencing, and other high-throughput functional genomics data [7, 8]. The integration and reanalysis of these data can provide valuable clues for COPD research, and hundreds of differentially expressed genes (DEGs) have been identified. However, the results remain inconsistent partly due to heterogeneity in independent studies, or the results are obtained from a single cohort study. Therefore, the integration of expression profiling data and bioinformatics techniques might overcome these limitations and may provide insight into novel molecular pathways underlying this heterogeneous disease. Microarray gene expression profiling of epithelial cells from the small airways with different smoking status provides a genome-wide assessment of molecular state and responses associated with COPD progression due to CS exposure.

In this study, after systematic searches of GEO datasets, 8 relevant studies comprising samples from 55 COPD smokers, 106 phenotypically normal smokers, and 78 normal nonsmokers were included. We have established CS-induced COPD rat models and performed transcriptomic analysis of lung specimens obtained after 0, 3, and 6 months of CS exposure and compared the results of gene expression with datasets obtained from human microarrays. In addition, we validated the expression of the selected genes by quantitative real-time polymerase chain reaction (qRT-PCR) in 16HBE with CS extract exposure over time, in CS-induced model rat lung tissues, and in human peripheral PBMC with different smoking status. Based on the overlapping gene panels, logistic regression analysis was carried out to establish discrimination models in response to CS.

We hypothesized that exposure to different degrees of CS might exhibit differences in gene expression. Potential DEG candidates could be identified that can accurately predict the disease progression in response to smoking.

## 2. Methods

### 2.1. Literature Searches and Biospecimen Collection

**Figure 1:**
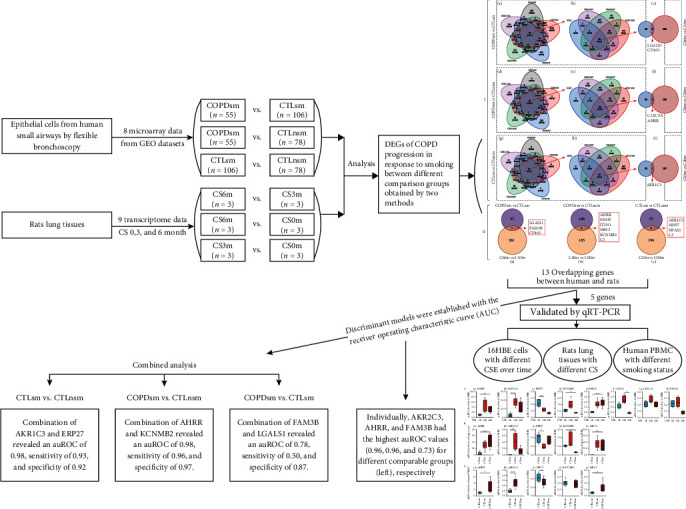
Study design. A total of 8 relevant studies comprising samples from 106 phenotypic normal smokers, 78 normal nonsmokers, and 55 COPD smokers based on GPL570 platform (Affymetrix Human Genome U133 Plus 2.0 Array) were included in this study. Epithelial cells from the small airways were sampled using flexible bronchoscopy. And corresponding rat lung tissues following 0, 3, and 6 months of CS exposure were obtained. To identify the DEGs during COPD progression, we compared DEGs between different groups, including COPD smokers vs. normal smokers, COPD smokers vs. normal nonsmokers, and normal smokers vs. normal nonsmokers. Similar CS exposure-induced COPD rat models were generated, and corresponding comparisons were made between rat with CS-exposure for 6 months (CS6m) vs. rat with CS-exposure for 3 months (CS3m), CS6m vs. rat exposed to normal air (CS0m), and CS3m vs. CS0m (CS6m represented COPD model; CS3m meant phenotypically normal smokers; CS0m as normal non-smokers).

The human microarray data were retrieved from the GEO database by systematic literature search using specific keywords strategy “COPD” and “epithelial cells” and “small airway” concatenated with “GPL570”, until Jan 2020. A total of 8 relevant studies [9–14] comprising samples from 106 phenotypic normal smokers, 78 normal nonsmokers, and 55 COPD smokers based on GPL570 platform (Affymetrix Human Genome U133 Plus 2.0 Array) were included in this study. All patients were smokers with established COPD (GOLD spirometry stages 0-3) who met the Global Initiative for Chronic Obstructive Lung Disease (GOLD) criteria [1], while controls had a normal medical history and physical examination and normal spirometry results. Normal nonsmokers were defined as never or former smokers with less than 5 pack-years. Normal smokers were defined as people who reported smoking currently [15]. Smokers were asked to abstain from smoking before the procedure. Demographic data on 8 human microarray was detailed in Table S1-S3. Additional 10 mild-to-moderate COPD, 11 normal smokers, and 11 normal nonsmokers for validation were also included. PBMCs were isolated using lymphocyte separation medium according to the method described previously and the detailed demographic data (Table S4), and study design is shown in Figure 1.

The study was conducted according to the criteria set by the Declaration of Helsinki, and written informed consent was obtained from all participants before data collection. The study was approved by the Institutional Review Board (GZMC 2009-08-1336).

### 2.2. Epithelial Cell Processing

Epithelial cells from the small airways were sampled using flexible bronchoscopy. Small airway samples were mostly collected from 10th to 12th order bronchi. Briefly, a 2 mm diameter brush was advanced approximately 7-10 cm distally from the 3rd order bronchus under fluoroscopic guidance. The distal end was compressed in a bronchus of comparable size in the right lower lobe, and cells were gently collected by careful brushing. Subsequently, the small airway epithelial cells were collected in proper medium. Total RNA was extracted from epithelial cells using TRIzol reagent (Life Technologies, Carlsbad, CA, United States) following manufacturer's protocol. Gene expression was analyzed with GeneChip® Human Genome U133 Plus 2.0 arrays (Affymetrix, Santa Clara, CA, USA).

### 2.3. Screening of Differentially Expressed Genes

To identify the DEGs during COPD progression, we compared DEGs between different groups, including COPD smokers vs. normal smokers, COPD smokers vs. normal nonsmokers, and normal smokers vs. normal nonsmokers. Similar CS exposure-induced COPD rat models were generated, and corresponding comparisons were made between rat with CS-exposure for 6 months (CS6m) vs. rat with CS-exposure for 3 months (CS3m), CS6m vs. rat exposed to normal air (CS0m), and CS3m vs. CS0m (CS6m represented COPD model; CS3m meant phenotypically normal smokers; CS0m as normal nonsmokers).

### 2.4. Smoke Exposure Chamber and Rat Models of COPD

The CS exposure-induced COPD rat models were established, as described previously [16]. Briefly, rat was kept in a whole-body exposure chamber and exposed to CS with 9 cigarettes/hour, 2 hours/exposure, twice/day for 6 days per week for a total of 3 or 6 months. Controls were kept in an identical facility and exposed to clean air.

For each experiment, rats were anesthetized with sodium pentobarbital (50 mg/kg body weight), and pulmonary function was assessed. Upon the onset of complete apnea, the lungs were immediately excised and weighed. The remaining whole left lung was used for histopathological analysis. The animals were cared for in accordance with the Guide for the Care and Use of Laboratory Animals in China (National Institutes of Health, China). All procedures and animal experiments were approved by the institutional review board of the Guangzhou Medical University.

### 2.5. Rat Pulmonary Function

Pulmonary function tests were carried out as described previously by Zhang et al. [17, 18]. The chord compliance (Cchord), total lung capacity (TLC), functional residual capacity (FRC), forced expiratory volume in 1 Ms (FEV_100_)/forced vital capacity (FVC), and inspiratory resistance (RI) were obtained according to the Buxco resistance/compliance application manual.

### 2.6. Rat Histopathology

For histopathological examination, the left whole lung was infused, washed with phosphate buffered saline, fixed with 4% paraformaldehyde, dehydrated in graded alcohol, embedded in paraffin, sectioned at 4 *μ*m thickness, and stained with hematoxylin and eosin (H&E). All stained sections were analyzed by microscopy.

### 2.7. Transcriptome Sequencing Profiling of Rat Lung Tissue

For transcriptome analysis (Genesky Biotechnologies Inc., Shanghai, 201315), total RNA was extracted from rats' lung tissue samples, which were obtained from animals exposed to cigarette smoke for 3 months, 6 months, and corresponding controls using TRIzol reagent according to the manufacturer's instructions. RNA concentration and purity were assessed using NanoDrop-1000 (Thermo Fisher Scientific, Waltham, MA, USA) and 2100 Bioanalyzer (Agilent, Santa Clara, CA, USA). RNA-seq libraries were prepared using RNA Library Prep Kit for Illumina, followed by paired-end sequencing on the Illumina HiSeq 2500 sequencing platform with a 125 bp read length. The software integrated within the sequencer converts the raw sequencing signals into base calling and stored in the FASTQ file format.

### 2.8. Prediction of Target miRNAs of DEGs

The miRNA sequences were downloaded from the miRBase version 22. We predicted miRNA target binding sites in the genome. Using miRNA reference databases, miRanda [19], and TargetScan [20], network analyses of the DEG mRNAs and miRNAs were performed.

### 2.9. Cell Culture and Quantitative Real-Time PCR (RT-PCR)

16HBE were purchased from Cell Bank of the Chinese Academy of Sciences (Shanghai, China), and the *in vitro* experiment was performed as described previously by Zhang et al. [17]. Briefly, the 16HBE cells (5 × 10^3^ cells per well) were seeded in a six-well plate (Corning, Corning, NY, United States) and exposed to cigarette smoke extract (CSE; 2%) for 0 h, 24 h, and 48 h. Each experiment was repeated thrice.

Total RNA was extracted from 16HBE cells, rat lung tissues, and human PBMC using TRIzol reagent and reverse-transcribed into first-strand cDNA. Real-time PCR was performed with the cDNA using SYBR Green Fast qPCR mix (Takara) with an iCyler iQ Real-time PCR Detection System (Bio-Rad Laboratories Inc., USA). The 18s was used as an internal reference, and the relative gene expression as fold change was calculated using the 2^-*ΔΔ*CT^ method. All experiments were repeated three times for each gene. The target gene and their primer sequences were listed in Table S5.

### 2.10. Statistical Analysis

Categorical variables were presented as number (%) and compared using the chi-squared test. Normally or nonnormally distributed continuous variables were presented as mean (standard deviation (SD)) or as median (interquartile range (IQR)) and compared continuous using independent *t*-test or Mann–Whitney test, respectively. The Database for Annotation, Visualization, and Integrated Discovery (DAVID; http://david.abcc.ncifcrf.gov/) provides a comprehensive set of functional annotation tools for the biological interpretation of large, “interesting” lists of genes. Gene expression values of the |log_2_FC|¤1 and *P* < 0.01 were used for filtering DEGs. Logistic regression models were established using a stepwise method (with a variable entered and removed if *P* < 0.05 and *P* > 0.1, respectively). Models were then compared by generating area under the curve (AUC) of the receiver operating characteristic (ROC), and the most simplified model (with least parameters) and without significant loss of the AUC of the ROC (>0.80) adopted. All statistical analyses were performed with a standard software package (Stata, version.15; Stata Corp LLC, TX 77845, USA) and OriginLab 2020b Graphing and Data Analysis software (OriginLab Corporation, MA, USA). All tests were two-tailed, and the *P* value of < 0.05 was considered statistically significant. For qRT-PCR validation, *P* values were adjusted for multiple comparisons using the Bonferroni correction.

## 3. Results

### 3.1. Rat Models Exhibited Varying Degrees of COPD-Related Alterations following CS Exposure

**Figure 2:**
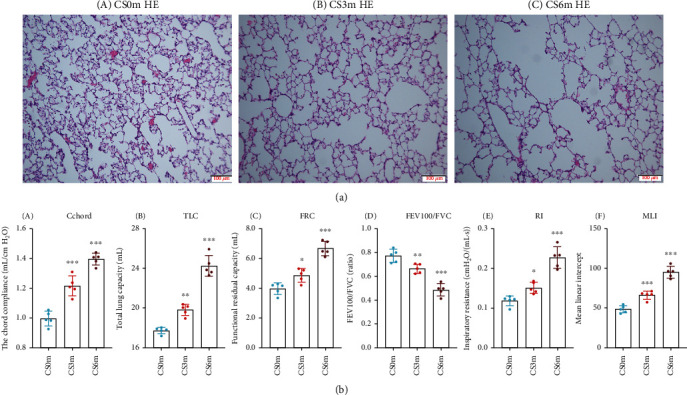
Lung histopathology and spirometry of the rat respiratory system response to CS exposure. (a) Lung histopathology from rat exposed to CS over time. (b) Rat spirometry indexes.

Measurements of lung histopathology and spirometry provided an overview of the overall response of the rat respiratory system to CS exposure. The effects of CS exposure were evident in H&E-stained lung tissue specimens.  Figure 2(a), A presents a histopathological examination of control rat at 0 months of CS exposure.  Figure 2(a), B and C illustrates histopathological changes following CS exposure for 3 and 6 months under the field of view at 100x magnification, respectively, and it showed fragmented and free-floating alveolar septa characteristic of a COPD-like phenotype in CS exposure for 6 months. CS resulted in a significant increase of mean linear intercept (MLI) at 3 and 6 months of CS exposure compared to ambient air rats. Dynamic spirometry was evaluated at 0, 3, and 6 months of CS exposure in rat. Compared to ambient air rats, the Cchord, TLC, FRC, and RI were significantly higher at 3 and 6 months of CS exposure (*P* < 0.05), and the FEV_100_/FVC% was significantly lower at 3 and 6 months of CS exposure (*P* < 0.05) in Figure 2(b).

### 3.2. Identification of the Overlapping DEGs between Human and Rat

COPD smokers, phenotypically normal smokers, and normal nonsmoker human airway epithelial cell gene expression profile datasets, including GSE5058, GSE5060, GSE8545, GSE20257, GSE19407, GSE11906, GSE11784, and GSE10006, were obtained from GEO database. In total, 239 individuals were identified by the microarray analysis. CS-induced rat models provided valuable insights into the pathogenesis of COPD [21]. For transcriptome sequencing, total RNA was extracted from rat's lung tissue samples obtained from animals exposed to smoke for 6 months and 3 months and nonexposed controls that corresponded to human COPD smokers, phenotypically normal smokers, and normal nonsmokers.

**Figure 3:**
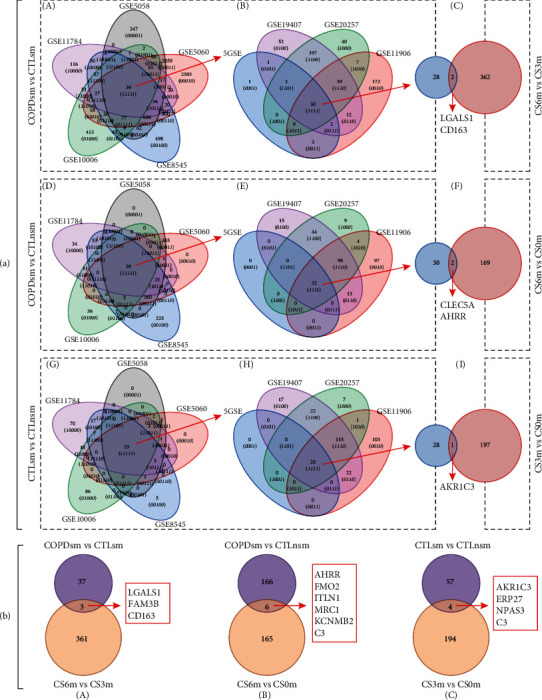
(a) Venn diagrams depicted the differentially expressed genes (DEGs) of overlapping between the gene expression profiles from each expression profile datasets of 8 human microarrays and rat exposed to CS over time. (a, A) The overlapping DEGs of 5 gene expression profiles from COPD smokers vs. normal smokers. (a, B) The overlapping DEGs of (a, A) and other 3 gene expression profiles from COPD smokers vs. normal smokers. (a, C) The overlapping DEGs between COPD smokers vs. normal smokers and rat with CS-exposure for 6 months (CS6m) vs. rat with CS-exposure for 3 months (CS3m). (a, D) The overlapping DEGs of 5 gene expression profiles from COPD smokers vs. normal nonsmokers. (a, E) The overlapping DEGs of (a, D) and other 3 gene expression profiles from COPD smokers vs. normal smokers. (a, F) The overlapping DEGs between COPD smokers vs. normal nonsmokers and CS6m vs. CS0m. (a, G) The overlapping DEGs of 5 gene expression profiles from normal smokers vs. normal nonsmokers. (a, H) The overlapping DEGs of (a, G) and other 3 gene expression profiles from normal smokers vs. normal nonsmokers. (a, I) The overlapping DEGs between normal smokers vs. normal nonsmokers and CS3m vs. CS0m. (b) Venn diagrams depicted DEGs of overlapping between the gene expression profiles from the combined 8 human microarray and rat exposed to CS over time. (b, A) The overlapping DEGs between COPD smokers vs. normal smokers and CS6m vs. CS3m. (b, B) The overlapping DEGs between COPD smokers vs. normal nonsmokers and CS6m vs. CS0m. (b, C). The overlapping DEGs between normal smokers vs. normal nonsmokers and CS3m vs. CS0m. Each circle represents one study. The number in the circle indicates how many genes in these groups.

Using |log_2_FC|¤1 and *P* < 0.01 as cut-off criterion, two methods were used to identify target genes in human microarray data. First, we extracted common DEGs from each expression profile datasets. A total of 30, 32, and 29 consistently expressed genes were identified from the 8 profile datasets in COPD vs. normal smokers, COPD vs. normal nonsmokers, and normal smokers vs. normal nonsmokers, respectively (Figure 3(a)). Second, we extracted DEGs from the 8 combined datasets. After integrated bioinformatics analysis, a total of 40, 172, and 61 genes were identified from the combined expression profile datasets in COPD vs. normal smokers, COPD vs. normal nonsmokers, and normal smokers vs. normal nonsmokers, respectively (Figure 3(b)).

Similarly, two methods were also used to determine the overlapping genes between humans and rats. First, when compared the 30 common DEGs in COPD smokers vs. normal smokers with CS6m vs. CS3m, there were 2 overlapping genes between human and rats; when we compared the 32 common DEGs in COPD vs. normal nonsmokers with CS6m vs. nonexposed rats, 2 genes overlapping between them were identified. Next, when we compared the 29 common DEGs in normal smokers vs. normal nonsmokers with CS3m vs. nonexposed rats, only 1 overlapping gene between them was identified (Figure 3(a)). Second, we compared the 40 DEGs in COPD smokers vs. normal smokers with CS6m vs. CS3m; there were 3 overlapping genes between humans and rats. When we compared the 172 DEGs in COPD smokers vs. normal nonsmokers with CS6m vs. nonexposed rats, there were 6 overlapping genes between them. Similarly, when we compared the 61 DEGs in normal smokers vs. normal nonsmokers with CS3m vs. nonexposed rats, 4 overlapping genes were found between them (Figure 3(b)).

### 3.3. COPD Smokers vs. Normal Smokers and CS6m vs. CS3m

#### 3.3.1. Comparisons of Gene Expression Patterns

Two methods were involved: (1) to identify gene expression patterns in COPD, we separately extracted common DEGs from each of 8 human datasets and compared the final common DEG gene expression results with the rat lung transcriptomic profiles (Figure 3(a), C). The results indicated that there were 2 genes overlapping, also including CD163/Cd163, between humans and rats.

(2) To identify gene expression profiles in COPD, we compared the combined human microarray DEG gene expression results with the rat lung transcriptomic profiles. We hypothesized that rat models with airspace enlargement after 6 months of CS exposure (CS6m) might share similar gene expression pattern as COPD smokers, while CS exposure for 3 months (CS3m) may share similar molecular mechanisms as smokers without COPD. Therefore, we compared DEGs between COPD smokers vs. normal smokers with CS6m vs. CS3m (Figure 3(b), A). The results revealed that there were 3 significant overlapping genes between humans and rats; of these, one shared gene (CD163/Cd163) has been previously reported to be associated with COPD; besides, CD163/Cd163 was found to be downregulated in both the human COPD and rat gene datasets

#### 3.3.2. Enrichment Analyses of the Combined 8 Microarray Datasets

**Figure 4:**
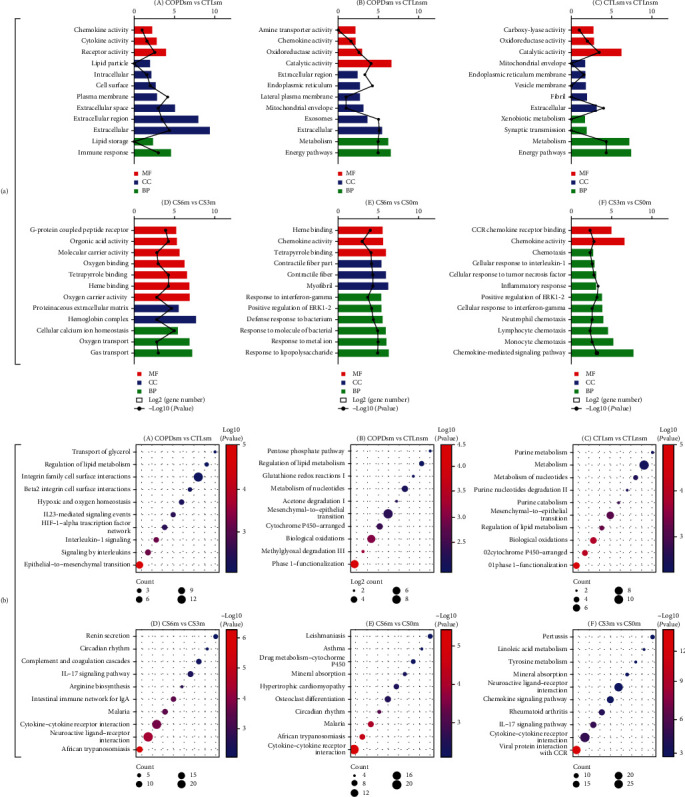
(a) Gene Ontology (GO) terms of the combined 8 human microarray and rat transcriptomic data. (A–C) Small airway epithelia cell differentially expressed gene (DEG) analysis. (D–F) Rat lung tissue DEG analysis. The rectangular length represents counts of the enriched DEGs. The line represents the negative log_2_*P* values (MF: molecular function; CC: cellular component; BP: biological process). (b) The Kyoto Encyclopedia of Genes and Genomes (KEGG) pathway enrichment of the combined 8 human microarray and rat transcriptomic data. (A–C) Small airway epithelia cell differentially expressed gene (DEG) analysis for different groups. (D–F) Rat lung tissue DEG analysis from rat with CS exposure for different groups. The dot sizes represent counts of the enriched DEGs. The dot colors represent the negative log_2_*P* value.

To investigate the functional roles of the above-mentioned DEGs, GO and KEGG analyses of significant DEGs were performed. The DEGs were classified into three functional groups: molecular function, biological process, and cellular component. In human datasets (Figure 4(a), A), from the enrichment results, we found that most of DEGs were significantly enriched in extracellular; in the biological process, genes predominantly enriched in immune response in COPD vs. normal smokers. Based on the molecular function, genes were mainly enriched in receptor activity and cytokine activity in COPD vs. normal smokers. In rat transcriptome sequencing datasets (Figure 4(a), D), the biological process analysis revealed that these genes were mainly distributed in cellular calcium ion homeostasis, oxygen transport, and gas transport function processes in CS6m vs. CS3m group. The molecular function analysis indicated that the genes were mainly enriched in organic acid/oxygen/tetrapyrrole/heme binding, G-protein coupled peptide receptor, and oxygen carrier activity. KEGG pathway analyses revealed that three pathways were enriched in COPD vs. normal smokers, including interleukin-1, interleukin signaling, and epithelial-to-mesenchymal transition (Figure 4(b), A); two pathways were enriched in CS6m vs. CS3m, including cytokine-cytokine receptor interaction and neuroactive-ligand receptor interaction (Figure 4(b), D).

**Figure 5:**
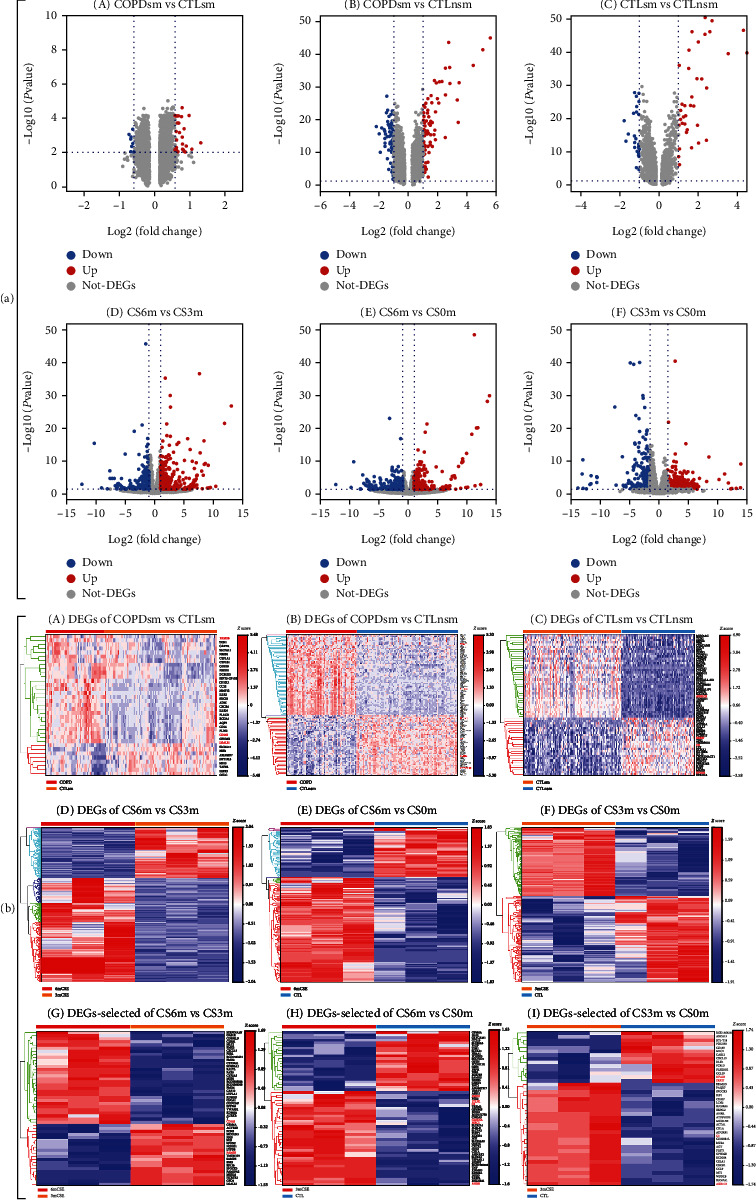
(a) The volcano plot of DEGs on the combined 8 human microarrays and rat transcriptomic data. (A–C) Volcano plot for DEGs in human small airway epithelia cell for different groups. (D–F) Volcano plot for DEGs in rat lung tissues from rat with CS exposure for different groups. Blue indicates genes with decreased expression, red indicates genes with increased expression, and white indicates genes with average expression. (b) Heat map of DEGs on the combined 8 human microarray and rat transcriptomic data. (A–C) Heat map for DEGs in human small airway epithelia cell for different groups, with the selected genes highlighted. (D–I) Heat map for DEGs in rat lung tissues from rat with CS exposure for different groups, with the selected genes highlighted. Each column represents a group, and each row represents a gene.

**Figure 6:**
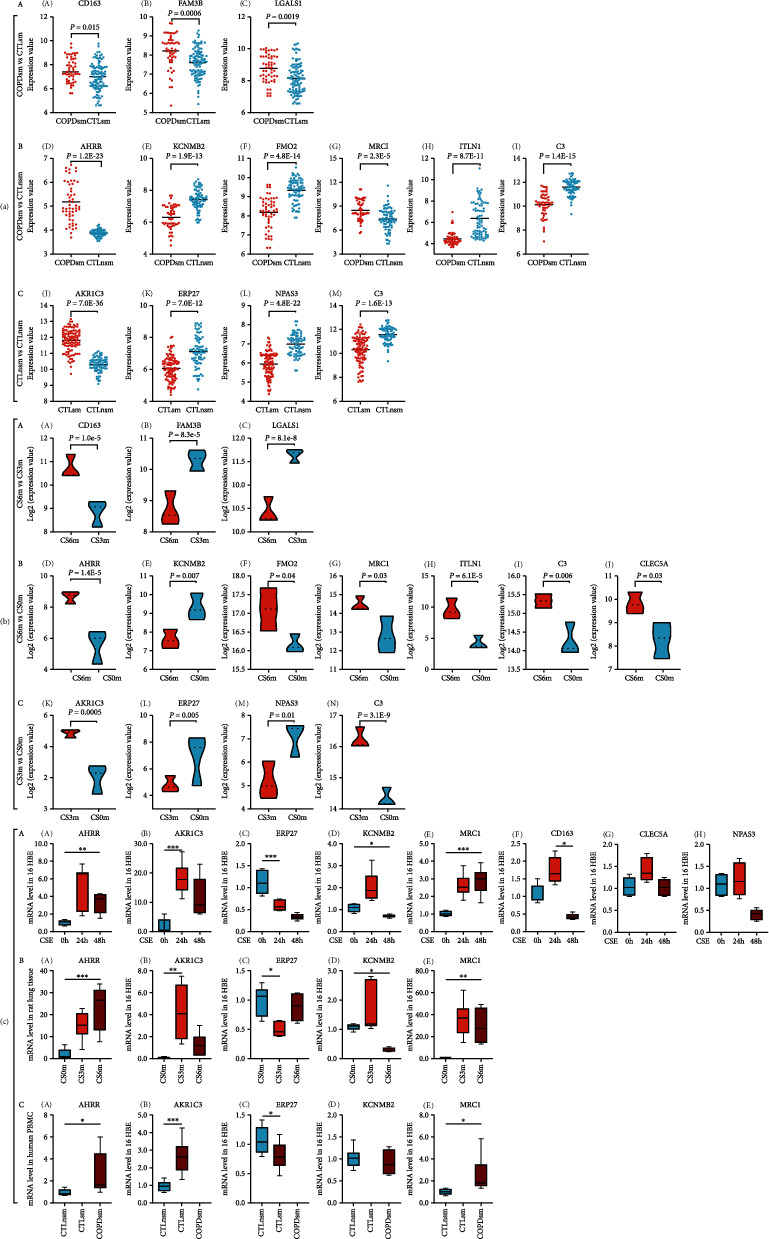
(a) The expressions of the selected DEGs in the combined 8 human microarrays. The horizontal axis represents groups, while the vertical axis for DEGs expressions. (b) The expressions of the selected DEGs in rat transcriptomic data. The horizontal axis represents groups, while the vertical axis for DEG expressions. (c) Effects of CSE on expression of selected genes validated in 16HBE, rat's lung tissues, and human PBMC by qRT-PCR.

Volcano plots of DEGs were generated to visualize the distribution of the expressed genes. Red or green dots in the plots represented significantly upregulated or downregulated genes, respectively.  Figure 5(a), A illustrates the DEGs among the combined 8 human microarrays in COPD vs. phenotypically normal smokers.  Figure 5(a), D represents the corresponding DEGs on rat transcriptomic data. As shown in Figure 5(b), heat maps were generated based on the expression levels of DEGs in 8 human GEO datasets and corresponding rat transcriptomic data.  Figure 5(b), A represents the combined analysis of 8 human microarray datasets in COPD vs. normal smokers; Figures 5(b), D and 5(b), G represent the corresponding rat transcriptomic data in CS6m vs. CS3m with the overlapping genes highlighted in red color. The significant overlapping genes (|log_2_FC|¤1 and *P* < 0.01) between COPD smokers vs. normal smokers and CS6m vs. CS3m included CD163/Cd163, FAM3/Fam3, and LGALS1/Lgals1 (also shown in Figures 6(a), A and 6(b), A).

#### 3.3.3. Enrichment Analyses of the 8 Microarray Data Separately

In the biological process, the common DEGs were mainly enriched in immune response (6/8) and cell communication (3/8) in COPD vs. normal smokers. In the molecular function, genes mainly enriched in receptor activity (6/8) and cytokine activity (3/8). Most of them existed in extracellular (6/8) and extracellular space (6/8) (Figure S1). Using KEGG enrichment analyses, we found that there was one pathway enriched in epithelia-to-mesenchymal transition (Figure S4). Volcano plots presented the distribution of the shared DEGs from each expression profile dataset, and the overlapping genes included CD163/Cd163 and LGALS1/Lgals1 (Figure S7, Figure S10A).

### 3.4. COPD Smokers vs. Normal Nonsmokers and CS6m vs. CS0m

#### 3.4.1. Comparison of Gene Expression Profiles

Two methods were involved: (1) we compared gene expression patterns upon COPD and smoking interaction; we separately extracted common DEGs from each human dataset and compared the final DEG gene expression results with the rat lung transcriptomic profiles (Figure 3(a), F). The findings revealed that there were 2 overlapping genes between human and rat datasets, including AHRR/Ahrr and CLEC5A/Clec5a.

(2) To compare gene patterns upon COPD and smoking interaction, we also compared the individual gene expression results with transcriptomic profiles of the rat. We hypothesized that normal nonexposed rats might share similar molecular mechanisms with normal nonsmokers. Therefore, we compared DEGs between COPD vs. normal nonsmokers with CS6m vs. normal nonexposed rats (Figure 3(b), B). The results indicated that there were 6 overlapping genes between humans and rats; of these, 3 genes were consistent direction in both the human COPD and rat gene datasets, including AHRR/Ahrr, KCNMB2/Kcnmb2, and MRC1/Mrc1

#### 3.4.2. Enrichment Analyses of the Combined Microarray Datasets

In the human dataset, GO analysis showed that most of DEGs were located in extracellular and exosomes (Figure 4(a), B); in the biological process, genes mainly enriched in metabolism and energy pathways in COPD vs. normal nonsmokers. In the molecular function, genes were mainly enriched in oxidoreductase activity and chemokine activity in COPD vs. normal nonsmokers. In the rat transcriptome sequencing dataset (Figure 4(a), E), the biological process analysis revealed that these genes were mainly distributed in bacterial molecules, metal ion, lipopolysaccharide, and bacterium function processes in CS6m vs. nonexposed group. The molecular function analysis indicated that DEGs were mainly distributed in heme/tetrapyrrole binding and chemokine activity. KEGG pathway enrichment analyses revealed that there were two pathways enriched in COPD vs. nonsmokers, including biological oxidations and phase 1-functionalization (Figure 4(b), B); however, one pathway of cytokine-cytokine receptor interaction was enriched in CS6m vs. nonexposed rats (Figure 4(b), E).

Volcano plots showed that the DEGs among the combined 8 human microarrays (Figure 5(a), B); Figure 5(a), E illustrates the corresponding DEGs on rat RNA-seq data. Heat maps were generated based on the expression levels of DEGs in 8 human GEO datasets and corresponding rat RNA-seq data.  Figure 5(b), B represents the combined 8 human microarray data in COPD vs. normal nonsmokers; Figures 5(b), E and 5(b), H represent the corresponding rat RNA-seq data on CS6m vs. normal nonexposed rats with the overlapping genes highlighted in red color. The common significantly overlapping genes (|log_2_FC|¤1 and *P* < 0.01) between COPD vs. normal nonsmokers and CS6m vs. normal nonexposed were AHRR/Ahrr, KCNMB2/Kcnmb2, MRC1/Mrc1, FMO2/Fmo2, ITLN1/Itln1, and C3/c3 (also shown in Figures 6(a), B and 6(b), B).

#### 3.4.3. Enrichment Analyses of the 8 Microarray Datasets Separately

In the biological process, the common DEGs were mainly enriched in energy pathways (7/8) and metabolism (7/8) in COPD vs. normal nonsmokers. In the molecular function, genes enriched primarily on catalytic activity (7/8) and receptor activity (3/8); most of DEGs existed in extracellular (6/8) and extracellular space (5/8) (Figure S2). KEGG enrichment analyses revealed that only one pathway of epithelia-to-the mesenchymal transition was enriched (7/8, Figure S5). Volcano plots showed the distribution of the DEGs from each expression profile dataset, and the significant overlapping genes included AHRR/Ahrr and CLEC5A/Clec5a (Figure S8, Figure S10B).

### 3.5. Normal Smokers vs. Normal Nonsmokers and CS3m vs. Nonexposed Rats

#### 3.5.1. Comparisons of Gene Expression Profiles

Two methods were involved: (1) to compare gene patterns upon CS exposure, we separately extracted common DEGs from each human dataset and compared the final DEG gene expression results with the rat lung transcriptomic profiles (Figure 3(a), I). We found that there was one overlapping gene (AKR1C3/Akr1c3) between humans and rats also involved.

(2) To identify gene expression patterns upon CS exposure, we compared the combined human microarray results with the rat lung transcriptomic profiles. We compared DEGs between normal smokers vs. normal nonsmokers with CS3m vs. normal nonexposed (Figure 3(b), C). The results revealed that there were 4 significant overlapping genes between human and rats, and 3 genes, including AKR1C3/Akr1c3, ERP27/Erp27, and NPAS3/Npas3, were consistent in both the human COPD and rat gene expression datasets

#### 3.5.2. Enrichment Analyses of the Combined 8 Human Microarray Datasets

In the human dataset, GO analysis showed that most of DEGs were located in extracellular (Figure 4(a), C); in the biological process, genes mainly enriched in metabolism and energy pathways in normal smokers vs. nonsmokers. In the molecular function, genes were mainly enriched in catalytic activity in normal smokers vs. nonsmokers. In the rat transcriptome sequencing dataset (Figure 4(a), F), the biological process analysis revealed that these genes were mainly distributed in the chemokine-mediated signaling pathway in CS3m vs. nonexposed group. The molecular function analysis indicated that they were mainly distributed in chemokine activity. KEGG pathway analyses demonstrated that two pathways, including biological oxidations and phase 1-functionalization, were enriched in normal smokers vs. nonsmokers (Figure 4(b), C); one pathway of cytokine-cytokine receptor interaction was enriched in CS3m vs. nonexposed rats (Figure 4(b), F).

Volcano plots revealed DEGs among the combined 8 human microarrays in normal smokers vs. normal nonsmokers (Figure 5(a), C); Figure 5(a), F shows the corresponding DEGs on rat RNA-seq data. Heat maps are presented in Figure 5(b), C that illustrated the combined human microarray datasets in normal smokers vs. normal nonsmokers; Figures 5(b), F and 5(b), I represent the corresponding rat RNA-seq data with the overlapping genes highlighted in red color. The overlapping significantly genes (|log_2_FC|¤1 and *P* < 0.01) between normal smokers vs. normal nonsmokers and CS3m vs. nonexposed were AKR1C3/Akr1c3, ERP27/Erp27, NPAS3/Npas3, and C3/c3 (also shown in Figures 6(a), C and 6(b), C).

#### 3.5.3. Enrichment Analyses of the 8 Human Microarray Datasets Separately

In the biological process, the DEGs mainly enriched in energy pathways (8/8) and metabolism (8/8) in normal smokers vs. normal nonsmokers; in the molecular function, genes mainly enriched in catalytic activity (8/8), transferase activity, transferring (7/8), and oxidoreductase activity (7/8); most of them existed in extracellular (6/8) and extracellular space (5/8) (Figure S3). KEGG pathway analyses revealed that three pathways, including biological oxidations (5/8), functionalization (5/8), and cytochrome P450-arranged, were enriched (5/8, Figure S6). Volcano plots showed the distribution of the DEGs from each expression profile dataset, and the significant overlapping genes included AKR1C3/Akr1c3 (Figure S9, Figure S10C).

### 3.6. Validation of CS Exposure on Expression of Selected Genes

Seven overlapping genes were consistently identified in both the human microarray and rat gene datasets. These selected genes were further verified in 16HBE cells, rats' lung tissues, and human PBMC. It showed that four genes (AKR1C3/Akr1c3, ERP27/Erp27, AHRR/Ahrr, and MRC1/Mrc1) were consistently validated through qRT-PCR. For KCNMB2/Kcnmb2, it also showed significant differences between groups except the expression in the present limited PBMC (Figure 6(c)).

### 3.7. Prediction of Target miRNAs of DEGs

Finally, five DEGs were selected as the target genes. To analyze the interactions between miRNAs and DEGs, the network between differentially expressed miRNAs and target DEGs was analyzed by Cytoscape (Figure S11). We identified 4 DEGs associated with the miRNAs (Figure S11B, S11C, and S11E). Among them, 4 miRNAs, including hsa-mir-125b-5p, hsa-mir-124-3p, hsa-mir-18a-5p, and hsa-mir-26a-5p, had the target AHRR/Ahrr. The miRNAs, including hsa-mir-27a-3p and hsa-mir-4480, targeted the MRC1/Mrc1, while miRNAs, including hsa-mir-155-5p and hsa-mir-98-5p, targeted AKR1C3/Akr1c3. The miRNAs, including hsa-mir-574-5p, was found to target ERP27/Erp27. For other DEGs, no target miRNAs were identified (Figure S11A, S11D, and S11F).

### 3.8. Discriminant Model Establishment

**Figure 7:**
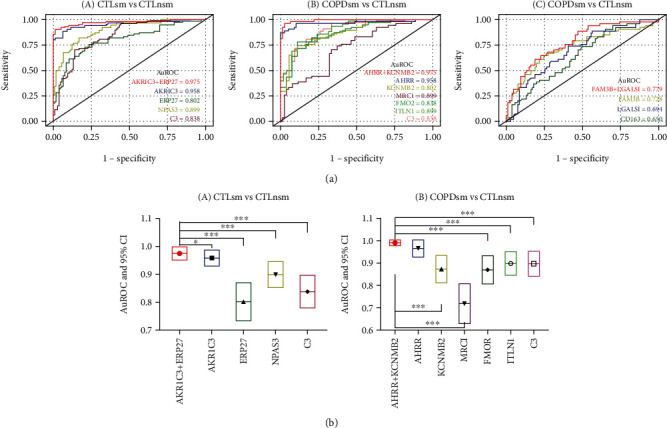
Efficacy prediction based on the overlapping genes panels. (A–C) Plots of ROC results for distinguishing CTL smokers from the CTL nonsmokers, for distinguishing COPD smokers from the CTL nonsmokers, and for distinguishing COPD smokers from the CTL smokers, respectively. The ROC curves were created by plotting the sensitivity (i.e., true positive rate) against 1-specificity (i.e., false positive rate). The blue line in each plot represents the area under the curve (AUC). (b) The corresponding statistical analysis.

We first explored the signatures of each gene profile separately, prior to multigene integration (Figure 7) and applied ROC models to calculate the auROC, specificity, and sensitivity of single gene. On the basis of the overlapping gene panels, 4, 6, and 3 discriminant models were established in normal smokers vs. normal nonsmokers, COPD smokers vs. normal nonsmokers, and COPD smokers vs. normal smoker groups, respectively. Results indicated that individually, AKR1C3/Akr1c3, AHRR/Ahrr, and FAM3/Fam3 had the highest auROC values (0.96, 0.96, and 0.73) for the above comparable groups, respectively (Table S6).

For analysis of predictive capability of combined genes, the combination of AKR1C3/Akr1c3 and ERP27/Erp27 revealed an auROC of 0.98, sensitivity of 0.93, and specificity of 0.92 in normal smokers vs. normal nonsmoker group. We used the same model to establish a logistic model using AHRR/Ahrr and KCNMB2/Kcnmb2, and COPD smoker participants can be distinguished predicted with high sensitivity (0.96) and specificity (0.97), and the auROC also reached 0.98 in our dataset, compared to normal nonsmokers (Figure 7). For COPD smokers vs. normal smoker groups, the combination of FAM3/Fam3 and LGALS1/Lgals1 revealed an auROC of 0.78, sensitivity of 0.50, and specificity of 0.87.

## 4. Discussion

The present study is the first to compare gene expression patterns of human small airway epithelial samples exposed to tobacco smoke over time with or without COPD and with that of rats' lungs following chronic CS exposure. Five consistently dysregulated DEGs were further experimentally validated, including AKR1C3/Akr1c3, ERP27/Erp27, AHRR/Ahrr, KCNMB2/Kcnmb2, and MRC1/Mrc1. Of these, we identified three genes that had not been reported previously, including ERP27/Erp27, KCNMB2/Kcnmb2, and MRC1/Mrc1. On the basis of the overlapping genes panel, effective and convenient discriminant models were established that can accurately predict the disease progression in response to different levels of smoking.

Altered expression of the antioxidant gene and oxidative damage related gene is a common response associated with CS exposure or COPD, such as AKR1C3. Its expression was significantly increased in normal smokers and CS exposed rats compared to the nonexposed group. AKR1C3, a type 5 17-*β*-hydroxy steroid dehydrogenase, is involved in the metabolism of potent trans-dihydrodiols containing more than two rings [22]. The enzyme has also been shown to oxidize polycyclic aromatic hydrocarbons (PAHs) to catechol, which can produce adione that can form stable and depurinating DNA adducts and/or ROS, leading to oxidative stress and oxidative DNA damage [23]. In addition, epigenome-wide association studies have revealed alterations in the DNA methylation status of candidate genes associated with cigarette smoking that are involved in the etiology of smoking-related diseases. DNA methylation at CpG sites of aryl hydrocarbon receptor repressor (AHRR) has been found to be associated with CS and lung function levels [24], indicating that AHRR DNA methylation could influence smoking dependence [25].

Smoking-related gene, ERP27, a noncatalytic member of the protein disulfide isomerase (PDI) family of endoplasmic reticulum (ER) proteins, is localized in the ER lumen that selectively binds unfolded or partially folded polypeptides [26, 27]. ERP27 comprises of two domains that are homologous to the noncatalytic *b* and *b*′ domains of PDI; however, it does not have the CXXC active site and hence is unable to catalyze dithiol-disulfide exchange [27]. It is likely to participate in protein folding and is involved in interactions with the disulfide isomerase ERP57 [26, 27]. Notably, difficult-to-express proteins (DEPs) are prone to misfold, and prolonged-expression of unfolded or misfolded proteins in the ER can lead to ER stress and activate the unfolded protein response (UPR) in the cell [28]. Thus, ERP27 and ERP57 overexpressions likely contribute directly to decrease the accumulation of misfolded DEPs, thereby preventing or delaying UPR-induced apoptosis. Moreover, ERP27 has also been identified to be downregulated in acute pancreatitis in rats [29] and exhibited extensive cytoplasmic expression in acinar cells of the human pancreas. In the present study, the level of ERP27 expression was revealed to be reduced in normal smokers and in CS3m compared to nonsmokers or nonexposed rats. This may explain the increased accumulation of misfolded DEPs and apoptosis in response to smoking.

Smoking and COPD phenotype interacting gene, KCNMB2 (potassium large-conductance calcium-activated channel, subfamily M, beta member 2), a potassium channel gene, it encodes the *β*-subunits of the large-conductance K^+^ channels, which is important for the control of smooth muscle tone and neuronal excitability. However, coexpression of KCNMB2 with KCNMA1 (potassium large conductance calcium-activated channel, subfamily M, alpha member 1) results in rapid and complete in activation of activating currents [30]. It was also found to confer rapid and complete inactivation to the BK channel (the large conductance calcium-sensitive potassium channel) complex and mediate inactivation gating [31]. Hypoxic pulmonary hypertension (HPH) is a frequently reported complication in COPD patients. ROS, hypoxia-inducible factors (HIF), and potassium channels (KV) are considered as the main factors driving the development of HPH. Hypoxia has been attributed to the production of ROS, which affects the balance of K^+^, followed by HIF system stabilization. Importantly, hypoxia-induced pulmonary vasoconstriction is dependent on the inhibition of KV channel function [32]. In the present study, KCNMB2 expression level was markedly reduced in CS exposure-induced COPD rat models and COPD smoker patients and may be attributed in promoting pulmonary vasoconstriction and arterial remodeling. Yet, another gene mannose receptor C-type 1 (MRC1), encoding the human mannose receptor (MR) related with innate immune responses, is a member of the pattern recognition C-type lectin receptors (CLR) family. Quantitative trait locus analysis demonstrated that MRC1 is associated with bronchial hyperresponsiveness [33]. Accumulating studies have also revealed that the MRC1 has been associated with increased susceptibility to asthma [34] and pulmonary tuberculosis [35]. This evidence may provide experimental and theoretical foundations to clarify the pathogenesis of COPD.

Furthermore, it has been well established that smoking affects gene expression patterns. Mild-to-moderate COPD smokers have different gene expression levels compared to phenotypic normal smokers and normal nonsmokers, while phenotypic normal smokers tend to have intermediate gene expression levels, depending on the time over smoking. Smoking influences expressions of specific genes, which contribute to the trajectory of COPD disease progress in response to smoking. In addition, we identified that the combined human microarray datasets as one dataset allowed us to identify additional DEGs and offer new insights into the mechanism underlying COPD that might have been missed when these datasets were analyzed separately.

This study has several limitations. First, the sample size of this study was relatively small and lacked further functional validations. Second, low level of overlap between the CS-induced COPD rat models and that of COPD patients, except the heterogeneity between human and the rat models, incomplete orthology between human and rat genes may play pivotal roles in it. Third, factors, including differences in human small airway epithelial cells and rat lung tissue samples and differences between the experimental CS exposure and human cigarette smoking [21, 36], may be attributed to exposure model serves as the gold standard model for COPD development; it fails to completely replicate the phenotype of chronic bronchitis and mucus overproduction in humans. Thus, CS-induced COPD in rat captures only a fraction of the COPD phenotypic spectrum. Lastly, the small airways are the earliest site of morphologic changes in COPD, and that progression of COPD is strongly associated with local changes in the small airways; however, for rats, these gene expression data were obtained from homogenized lung tissues, and they represent the average expression from mixtures of cell types.

## 5. Conclusion

The present study identified 5 DEG candidates of COPD progression in response to smoking and developed effective and convenient discriminant models that can accurately predict the disease progression. Although further studies are warranted, these candidate genes and pathways could serve as potential therapeutic targets to prevent the onset and progression of COPD.

## Data Availability

The data that support the findings of this study are available from the corresponding authors upon reasonable request.

## Abbreviations

CS: Cigarette smoking
CSE: Cigarette smoke extract
DEGs: Differentially expressed genes
COPD: Chronic obstructive pulmonary disease
GWAS: Genome-wide association studies
NCBI: National Center of Biotechnology Information
GEO: Gene Expression Omnibus
qRT-PCR: Quantitative real-time polymerase chain reaction
GOLD: Chronic obstructive lung disease
CS6m: Smoke exposure for 6 months
CS3m: Smoke exposure for 3 months
TLC: Total lung capacity
Cchord: The chord compliance
FRC: Functional residual capacity
FVC: Forced vital capacity
RI: Inspiratory resistance
GO: Gene Ontology
KEGG: Kyoto Encyclopedia of Genes and Genomes
FAM3/Fam3: Family with sequence similarity 3
AHRR/Ahrr: Aryl hydrocarbon receptor
KCNMB2/Kcnmb2: Ca^2+^-activated K^+^ channel (Kca1.1) *Β*2-subunit
FMO2/Fmo2: Flavin-containing monooxygenase 2
ITLN1/Itln1: Intelectin 1
AKR1C3/Akr1c3: Aldo-keto reductase 1 C3
ERP27/Erp27: Endoplasmic reticulum protein 27
NPAS3/Npas3: Neuronal PAS domain protein 3
MLI: Mean linear intercept.

## Supplementary Materials

Supplementary 1 Figure S1: Gene Ontology (GO) terms of the 8 separate microarrays in COPD smokers vs. normal smokers. The rectangular length represents counts of the enriched DEGs. The line represents the negative log_2_*P* values (MF: molecular function; CC: cellular component; BP: biological process). Figure S2: Gene Ontology (GO) terms of the 8 separate microarrays in COPD smokers vs. normal nonsmokers. The line represents the negative log_2_*P* values (MF: molecular function; CC: cellular component; BP: biological process). Figure S3: Gene Ontology (GO) terms of the 8 separate microarrays in normal smokers vs. normal nonsmokers. Figure S4: the Kyoto Encyclopedia of Genes and Genomes (KEGG) pathway enrichment of the 8 separate microarrays in COPD smokers vs. normal smokers. The dot sizes represent counts of the enriched DEGs. The dot colors represent the negative log_2_*P* value. Figure S5: the Kyoto Encyclopedia of Genes and Genomes (KEGG) pathway enrichment of the 8 separate microarray data in COPD smokers vs. normal nonsmokers. Figure S6: the Kyoto Encyclopedia of Genes and Genomes (KEGG) pathway enrichment of the 8 separate microarrays in normal smokers vs. normal nonsmokers. Figure S7: the volcano plot of DEGs on the 8 separate microarrays in COPD smokers vs. normal smokers. Blue indicates genes with decreased expression, red indicates genes with increased expression, and white indicates genes with average expression. Figure S8: the volcano plot of DEGs on the 8 separate microarrays in COPD smokers vs. normal nonsmokers. Figure S9: the volcano plot of DEGs on the 8 separate microarrays in normal smokers vs. normal nonsmokers. Figure S10: the expressions of the selected DEG expressions on the 8 separate microarrays. The horizontal axis represents groups, while the vertical axis for DEG expressions. Figure S11: the network between DEMs and differentially expressed miRNAs on the combined 8 human microarray and rat transcriptomic data. (A-C) The network for DEGs in human small airway epithelia cell from different groups. (D-F) The network prediction for DEGs in rat lung tissues from different groups.

Supplementary 2 Table S1: demographic data from 8 combined GEO datasets in GPL570. Table S2: demographic data from 8 single GEO datasets in GPL570. Table S3: detail demographic data from 8 GEO combined datasets. Table S4: demographic data of the validated participants. Table S5: primer sets used for real-time PCR. Table S6: predictive efficacy of single gene.
