# Supplementary material for: Gene Expression Trajectories from Normal Nonsmokers to COPD Smokers and Disease Progression Discriminant Modeling in Response to Cigarette Smoking
Source: Dis Markers. 2022 Sep 14;2022:9354286. doi: 10.1155/2022/9354286 (PMC9493146; doi:10.1155/2022/9354286)
Supplement: Supplementary 1 — Figure S1: Gene Ontology (GO) terms of the 8 separate microarrays in COPD smokers vs. normal smokers. The rectangular length represents counts of the enriched DEGs. The line represents the negative log2P values (MF: molecular function; CC: cellular component; BP: biological process). Figure S2: Gene Ontology (GO) terms of the 8 separate microarrays in COPD smokers vs. normal nonsmokers. The line represents the negative log2P values (MF: molecular function; CC: cellular component; BP: biological process). Figure S3: Gene Ontology (GO) terms of the 8 separate microarrays in normal smokers vs. normal nonsmokers. Figure S4: the Kyoto Encyclopedia of Genes and Genomes (KEGG) pathway enrichment of the 8 separate microarrays in COPD smokers vs. normal smokers. The dot sizes represent counts of the enriched DEGs. The dot colors represent the negative log2P value. Figure S5: the Kyoto Encyclopedia of Genes and Genomes (KEGG) pathway enrichment of the 8 separate microarray data in COPD smokers vs. normal nonsmokers. Figure S6: the Kyoto Encyclopedia of Genes and Genomes (KEGG) pathway enrichment of the 8 separate microarrays in normal smokers vs. normal nonsmokers. Figure S7: the volcano plot of DEGs on the 8 separate microarrays in COPD smokers vs. normal smokers. Blue indicates genes with decreased expression, red indicates genes with increased expression, and white indicates genes with average expression. Figure S8: the volcano plot of DEGs on the 8 separate microarrays in COPD smokers vs. normal nonsmokers. Figure S9: the volcano plot of DEGs on the 8 separate microarrays in normal smokers vs. normal nonsmokers. Figure S10: the expressions of the selected DEG expressions on the 8 separate microarrays. The horizontal axis represents groups, while the vertical axis for DEG expressions. Figure S11: the network between DEMs and differentially expressed miRNAs on the combined 8 human microarray and rat transcriptomic data. (A-C) The network for DEGs in human small airway epith [file 9354286.f1.zip › figures/Figure S2.pdf]

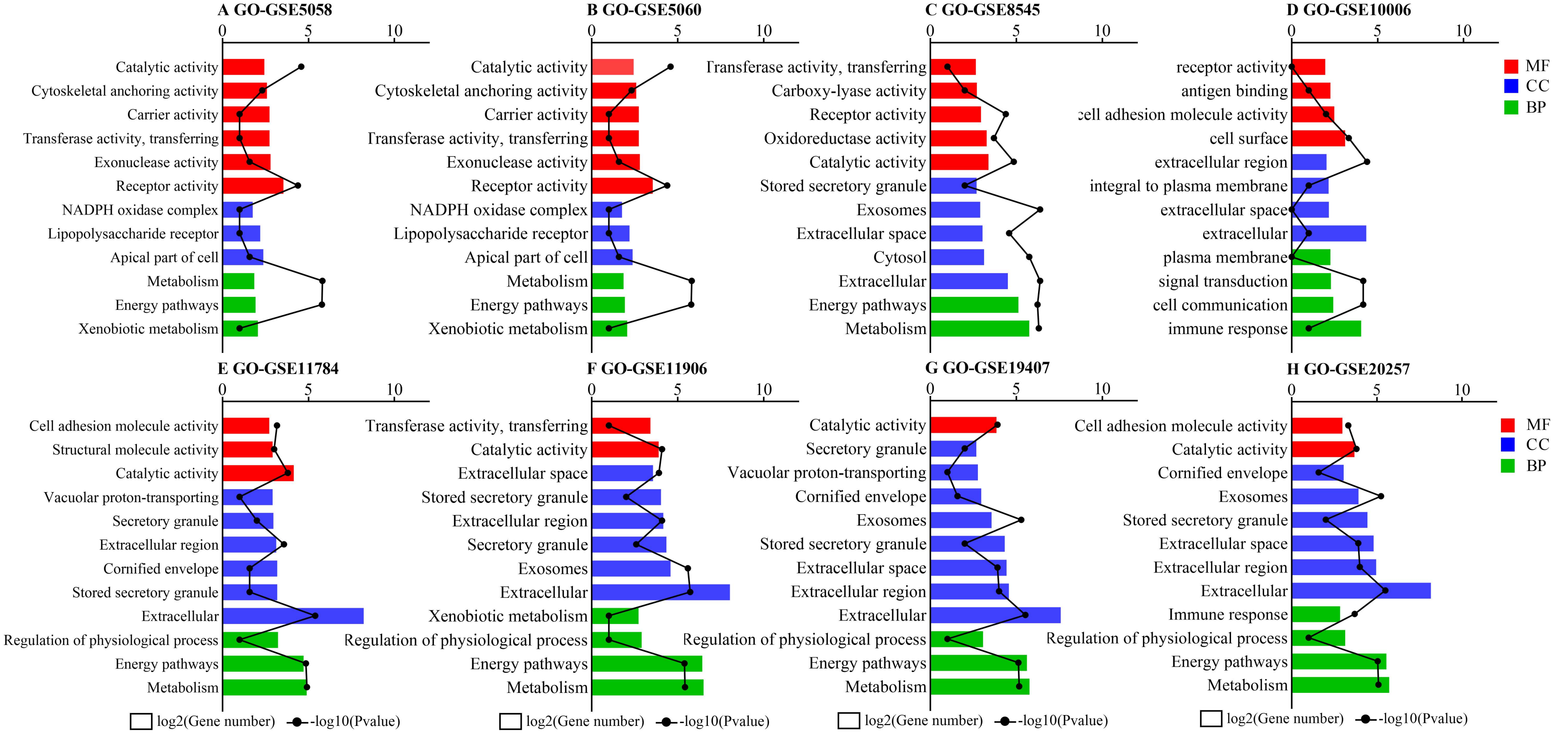

A GO-GSE5058
B GO-GSE5060
C GO-GSE8545
D GO-GSE10006
E GO-GSE11784
F GO-GSE11906
G GO-GSE19407
H GO-GSE20257
0
5
10
Catalytic activity
Cytoskeletal anchoring activity
Carrier activity
Transferase activity, transferring
Exonuclease activity
Receptor activity
NADPH oxidase complex
Lipopolysaccharide receptor
Apical part of cell
Metabolism
Energy pathways
Xenobiotic metabolism
Carboxy-lyase activity
Oxidoreductase activity
Stored secretory granule
Exosomes
Extracellular space
Cytosol
Extracellular
receptor activity
antigen binding
cell adhesion molecule activity
cell surface
extracellular region
integral to plasma membrane
extracellular space
extracellular
plasma membrane
signal transduction
cell communication
immune response
Cell adhesion molecule activity
Structural molecule activity
Vacuolar proton-transporting
Secretory granule
Extracellular region
Cornified envelope
Regulation of physiological process
Immune response
MF
CC
BP
log2(Gene number)
-log10(Pvalue)
