# Supplementary figures and images for: Gene Expression Trajectories from Normal Nonsmokers to COPD Smokers and Disease Progression Discriminant Modeling in Response to Cigarette Smoking
Source: Dis Markers. 2022 Sep 14;2022:9354286. doi: 10.1155/2022/9354286 (PMC9493146; doi:10.1155/2022/9354286)

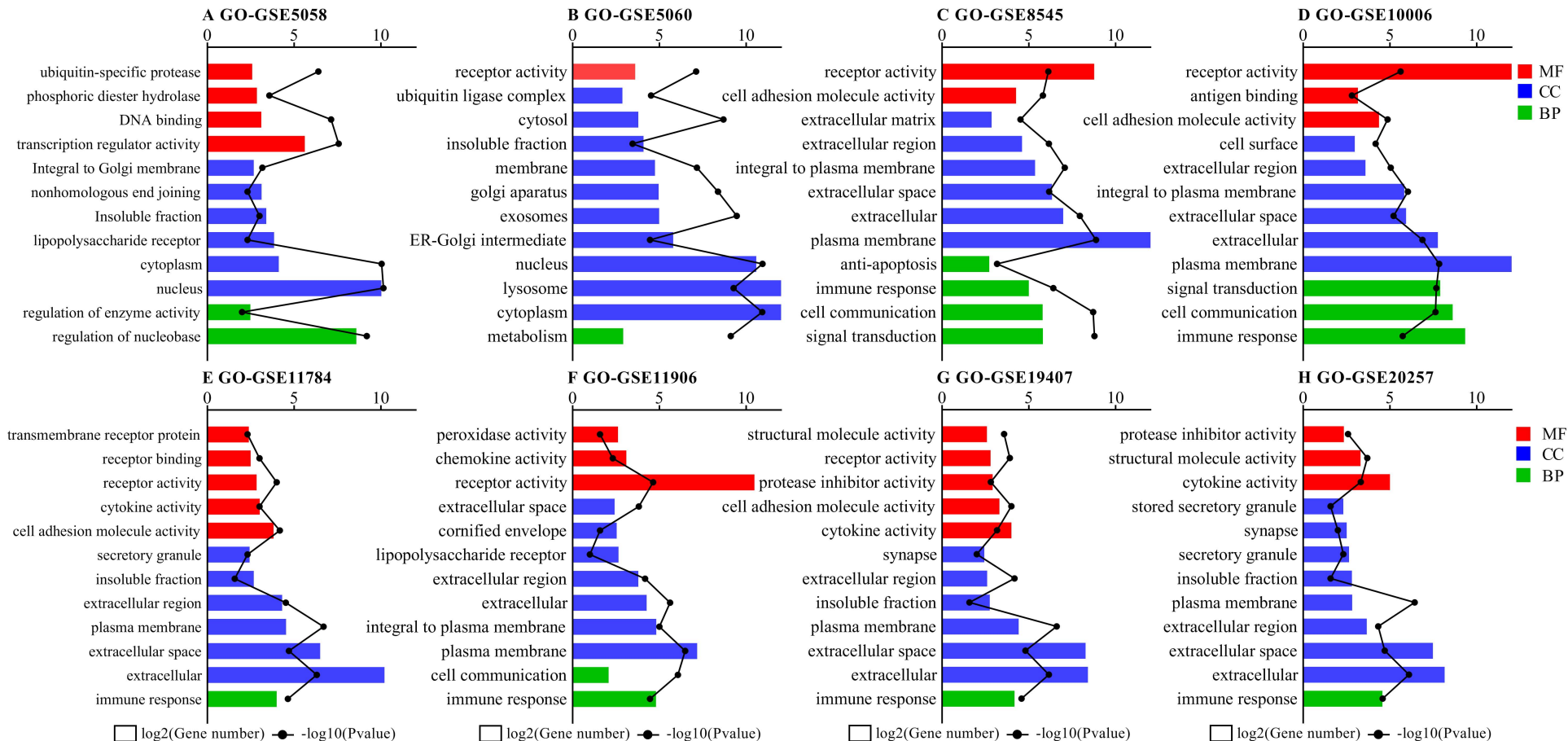

Supplement: Supplementary 1 — Figure S1: Gene Ontology (GO) terms of the 8 separate microarrays in COPD smokers vs. normal smokers. The rectangular length represents counts of the enriched DEGs. The line represents the negative log2P values (MF: molecular function; CC: cellular component; BP: biological process). Figure S2: Gene Ontology (GO) terms of the 8 separate microarrays in COPD smokers vs. normal nonsmokers. The line represents the negative log2P values (MF: molecular function; CC: cellular component; BP: biological process). Figure S3: Gene Ontology (GO) terms of the 8 separate microarrays in normal smokers vs. normal nonsmokers. Figure S4: the Kyoto Encyclopedia of Genes and Genomes (KEGG) pathway enrichment of the 8 separate microarrays in COPD smokers vs. normal smokers. The dot sizes represent counts of the enriched DEGs. The dot colors represent the negative log2P value. Figure S5: the Kyoto Encyclopedia of Genes and Genomes (KEGG) pathway enrichment of the 8 separate microarray data in COPD smokers vs. normal nonsmokers. Figure S6: the Kyoto Encyclopedia of Genes and Genomes (KEGG) pathway enrichment of the 8 separate microarrays in normal smokers vs. normal nonsmokers. Figure S7: the volcano plot of DEGs on the 8 separate microarrays in COPD smokers vs. normal smokers. Blue indicates genes with decreased expression, red indicates genes with increased expression, and white indicates genes with average expression. Figure S8: the volcano plot of DEGs on the 8 separate microarrays in COPD smokers vs. normal nonsmokers. Figure S9: the volcano plot of DEGs on the 8 separate microarrays in normal smokers vs. normal nonsmokers. Figure S10: the expressions of the selected DEG expressions on the 8 separate microarrays. The horizontal axis represents groups, while the vertical axis for DEG expressions. Figure S11: the network between DEMs and differentially expressed miRNAs on the combined 8 human microarray and rat transcriptomic data. (A-C) The network for DEGs in human small airway epith [file 9354286.f1.zip › figures/Figure S1.pdf]

**A****COPD vs CTLsm**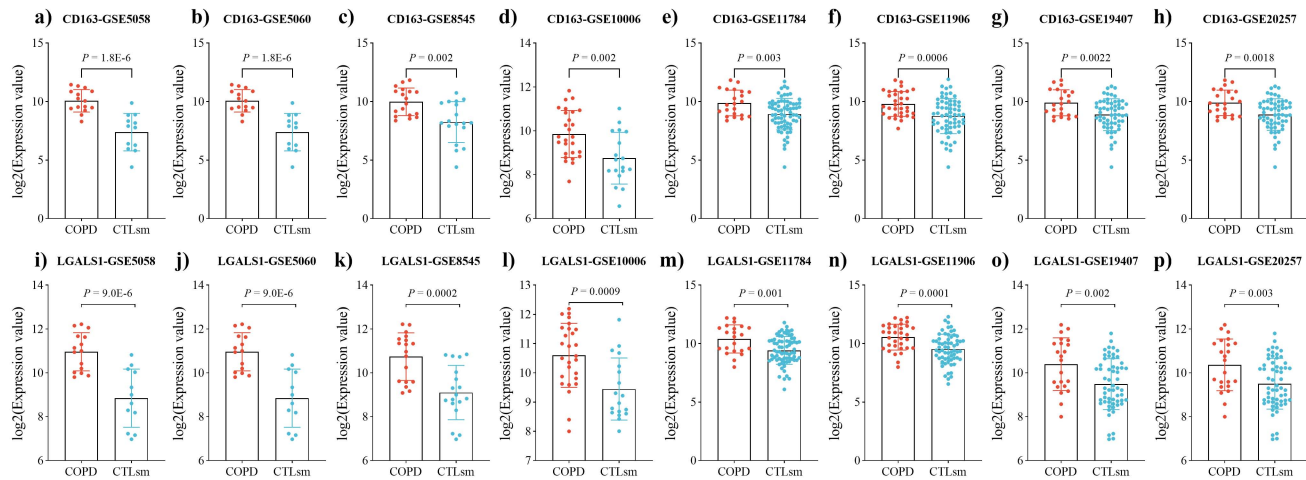**B****COPD vs CTLnsm**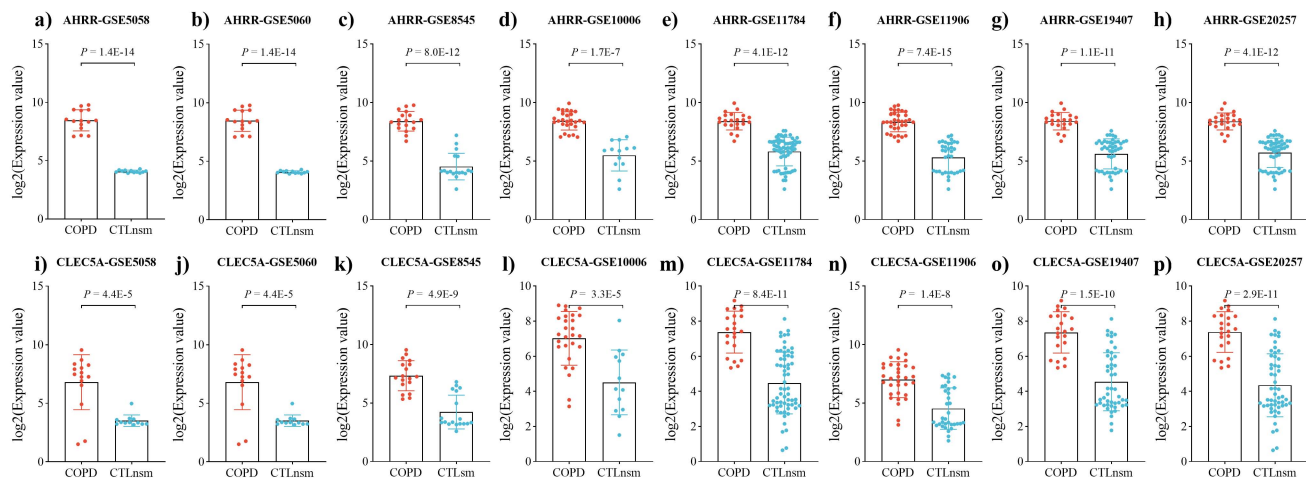**C****CTLsm vs CTLnsm**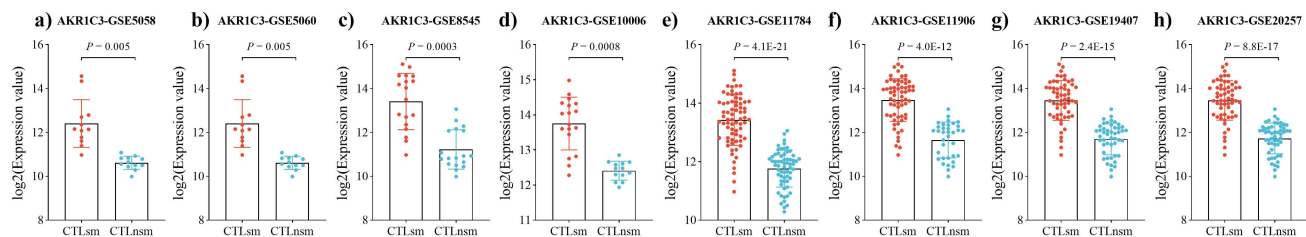

Supplement: Supplementary 1 — Figure S1: Gene Ontology (GO) terms of the 8 separate microarrays in COPD smokers vs. normal smokers. The rectangular length represents counts of the enriched DEGs. The line represents the negative log2P values (MF: molecular function; CC: cellular component; BP: biological process). Figure S2: Gene Ontology (GO) terms of the 8 separate microarrays in COPD smokers vs. normal nonsmokers. The line represents the negative log2P values (MF: molecular function; CC: cellular component; BP: biological process). Figure S3: Gene Ontology (GO) terms of the 8 separate microarrays in normal smokers vs. normal nonsmokers. Figure S4: the Kyoto Encyclopedia of Genes and Genomes (KEGG) pathway enrichment of the 8 separate microarrays in COPD smokers vs. normal smokers. The dot sizes represent counts of the enriched DEGs. The dot colors represent the negative log2P value. Figure S5: the Kyoto Encyclopedia of Genes and Genomes (KEGG) pathway enrichment of the 8 separate microarray data in COPD smokers vs. normal nonsmokers. Figure S6: the Kyoto Encyclopedia of Genes and Genomes (KEGG) pathway enrichment of the 8 separate microarrays in normal smokers vs. normal nonsmokers. Figure S7: the volcano plot of DEGs on the 8 separate microarrays in COPD smokers vs. normal smokers. Blue indicates genes with decreased expression, red indicates genes with increased expression, and white indicates genes with average expression. Figure S8: the volcano plot of DEGs on the 8 separate microarrays in COPD smokers vs. normal nonsmokers. Figure S9: the volcano plot of DEGs on the 8 separate microarrays in normal smokers vs. normal nonsmokers. Figure S10: the expressions of the selected DEG expressions on the 8 separate microarrays. The horizontal axis represents groups, while the vertical axis for DEG expressions. Figure S11: the network between DEMs and differentially expressed miRNAs on the combined 8 human microarray and rat transcriptomic data. (A-C) The network for DEGs in human small airway epith [file 9354286.f1.zip › figures/Figure S10.pdf]

**A. COPDsm vs CTLsm**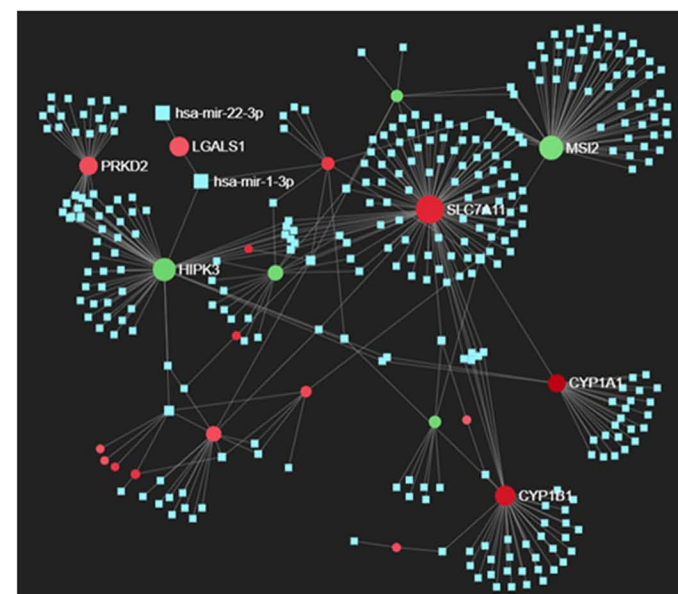**B. COPDsm vs CTLnsm**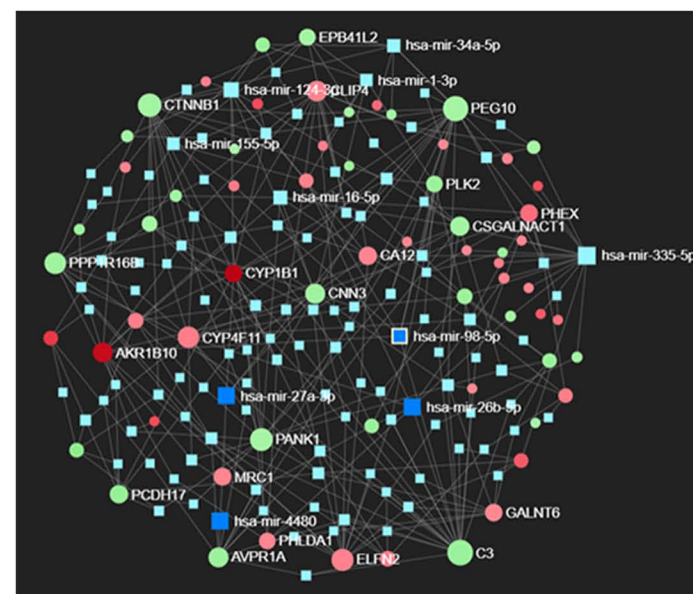**C. CTLsm vs CTLnsm**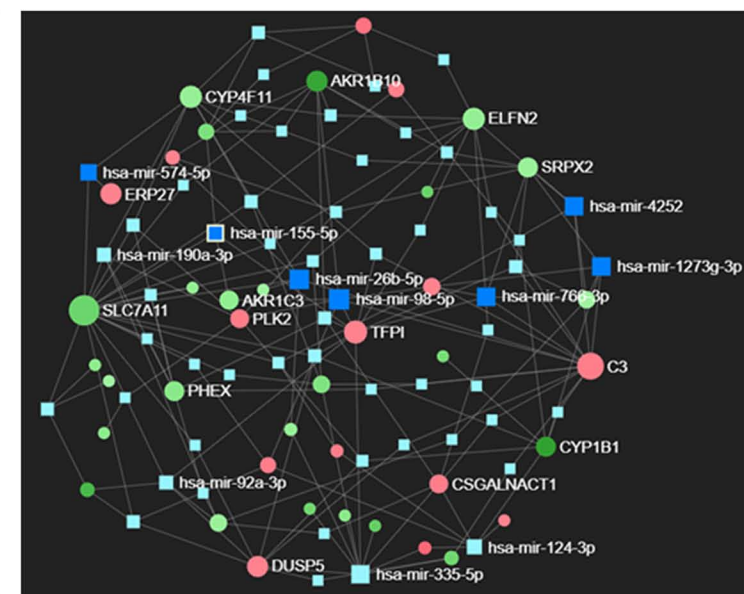**D. CS6m vs CS3m**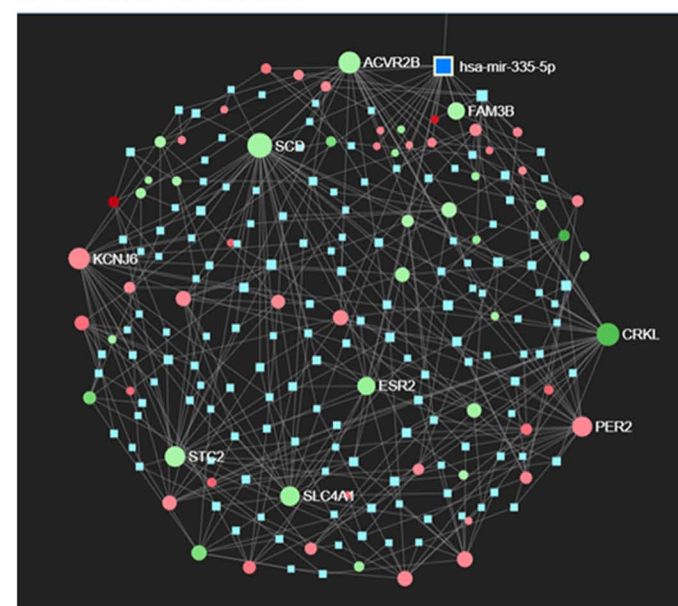**E. CS6m vs CS0m**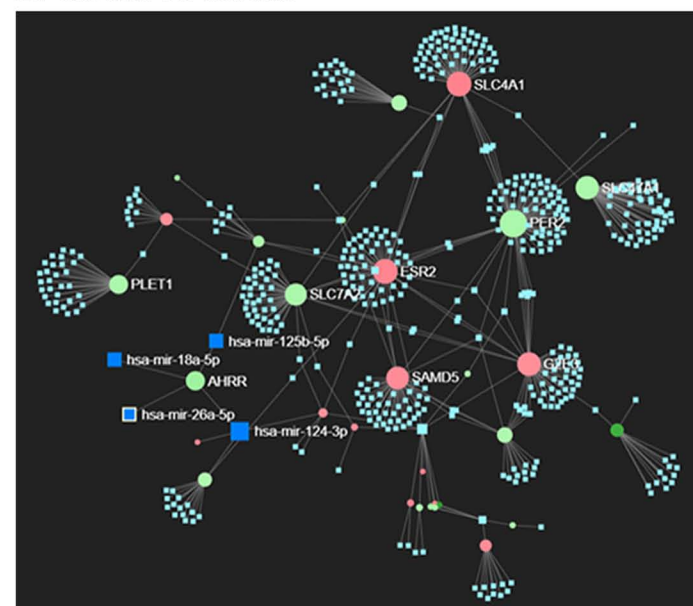**F. CS3m vs CS0m**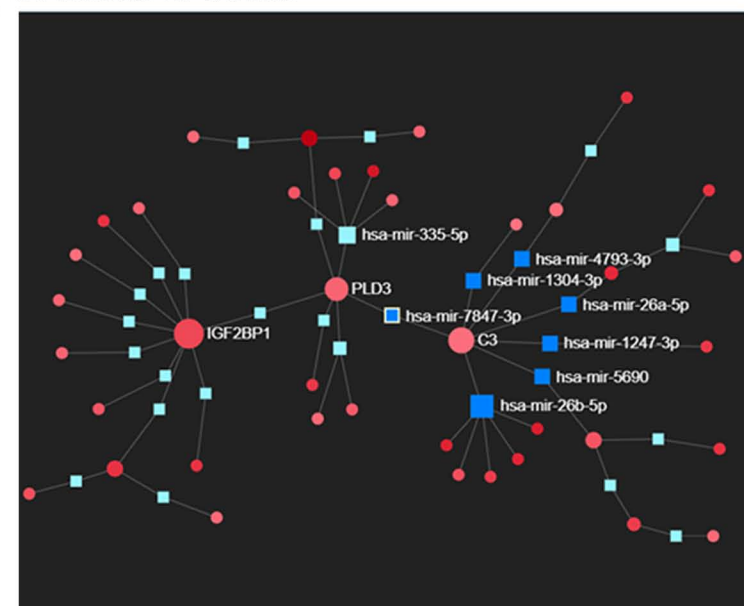

Supplement: Supplementary 1 — Figure S1: Gene Ontology (GO) terms of the 8 separate microarrays in COPD smokers vs. normal smokers. The rectangular length represents counts of the enriched DEGs. The line represents the negative log2P values (MF: molecular function; CC: cellular component; BP: biological process). Figure S2: Gene Ontology (GO) terms of the 8 separate microarrays in COPD smokers vs. normal nonsmokers. The line represents the negative log2P values (MF: molecular function; CC: cellular component; BP: biological process). Figure S3: Gene Ontology (GO) terms of the 8 separate microarrays in normal smokers vs. normal nonsmokers. Figure S4: the Kyoto Encyclopedia of Genes and Genomes (KEGG) pathway enrichment of the 8 separate microarrays in COPD smokers vs. normal smokers. The dot sizes represent counts of the enriched DEGs. The dot colors represent the negative log2P value. Figure S5: the Kyoto Encyclopedia of Genes and Genomes (KEGG) pathway enrichment of the 8 separate microarray data in COPD smokers vs. normal nonsmokers. Figure S6: the Kyoto Encyclopedia of Genes and Genomes (KEGG) pathway enrichment of the 8 separate microarrays in normal smokers vs. normal nonsmokers. Figure S7: the volcano plot of DEGs on the 8 separate microarrays in COPD smokers vs. normal smokers. Blue indicates genes with decreased expression, red indicates genes with increased expression, and white indicates genes with average expression. Figure S8: the volcano plot of DEGs on the 8 separate microarrays in COPD smokers vs. normal nonsmokers. Figure S9: the volcano plot of DEGs on the 8 separate microarrays in normal smokers vs. normal nonsmokers. Figure S10: the expressions of the selected DEG expressions on the 8 separate microarrays. The horizontal axis represents groups, while the vertical axis for DEG expressions. Figure S11: the network between DEMs and differentially expressed miRNAs on the combined 8 human microarray and rat transcriptomic data. (A-C) The network for DEGs in human small airway epith [file 9354286.f1.zip › figures/Figure S11-new.pdf]

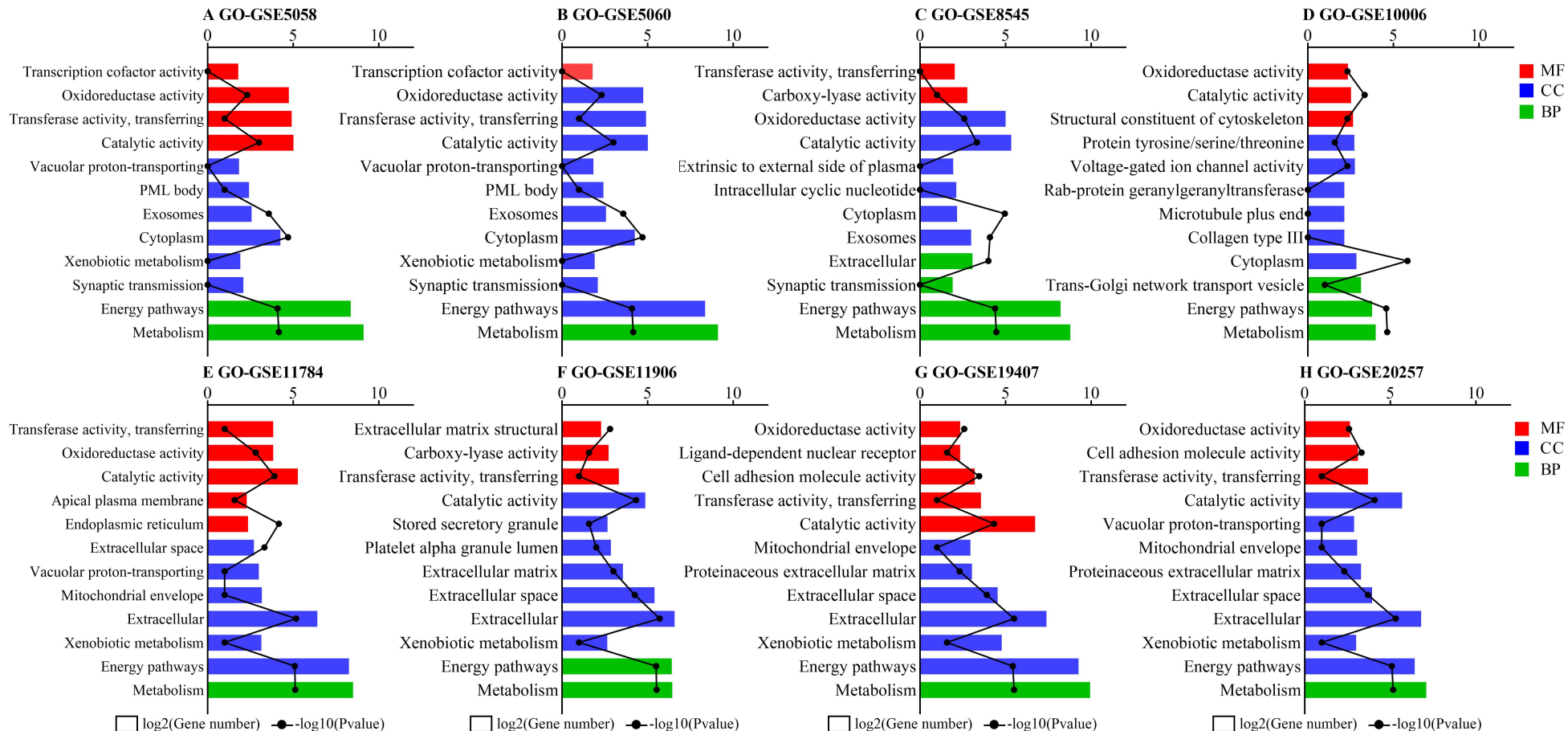

Supplement: Supplementary 1 — Figure S1: Gene Ontology (GO) terms of the 8 separate microarrays in COPD smokers vs. normal smokers. The rectangular length represents counts of the enriched DEGs. The line represents the negative log2P values (MF: molecular function; CC: cellular component; BP: biological process). Figure S2: Gene Ontology (GO) terms of the 8 separate microarrays in COPD smokers vs. normal nonsmokers. The line represents the negative log2P values (MF: molecular function; CC: cellular component; BP: biological process). Figure S3: Gene Ontology (GO) terms of the 8 separate microarrays in normal smokers vs. normal nonsmokers. Figure S4: the Kyoto Encyclopedia of Genes and Genomes (KEGG) pathway enrichment of the 8 separate microarrays in COPD smokers vs. normal smokers. The dot sizes represent counts of the enriched DEGs. The dot colors represent the negative log2P value. Figure S5: the Kyoto Encyclopedia of Genes and Genomes (KEGG) pathway enrichment of the 8 separate microarray data in COPD smokers vs. normal nonsmokers. Figure S6: the Kyoto Encyclopedia of Genes and Genomes (KEGG) pathway enrichment of the 8 separate microarrays in normal smokers vs. normal nonsmokers. Figure S7: the volcano plot of DEGs on the 8 separate microarrays in COPD smokers vs. normal smokers. Blue indicates genes with decreased expression, red indicates genes with increased expression, and white indicates genes with average expression. Figure S8: the volcano plot of DEGs on the 8 separate microarrays in COPD smokers vs. normal nonsmokers. Figure S9: the volcano plot of DEGs on the 8 separate microarrays in normal smokers vs. normal nonsmokers. Figure S10: the expressions of the selected DEG expressions on the 8 separate microarrays. The horizontal axis represents groups, while the vertical axis for DEG expressions. Figure S11: the network between DEMs and differentially expressed miRNAs on the combined 8 human microarray and rat transcriptomic data. (A-C) The network for DEGs in human small airway epith [file 9354286.f1.zip › figures/Figure S3.pdf]

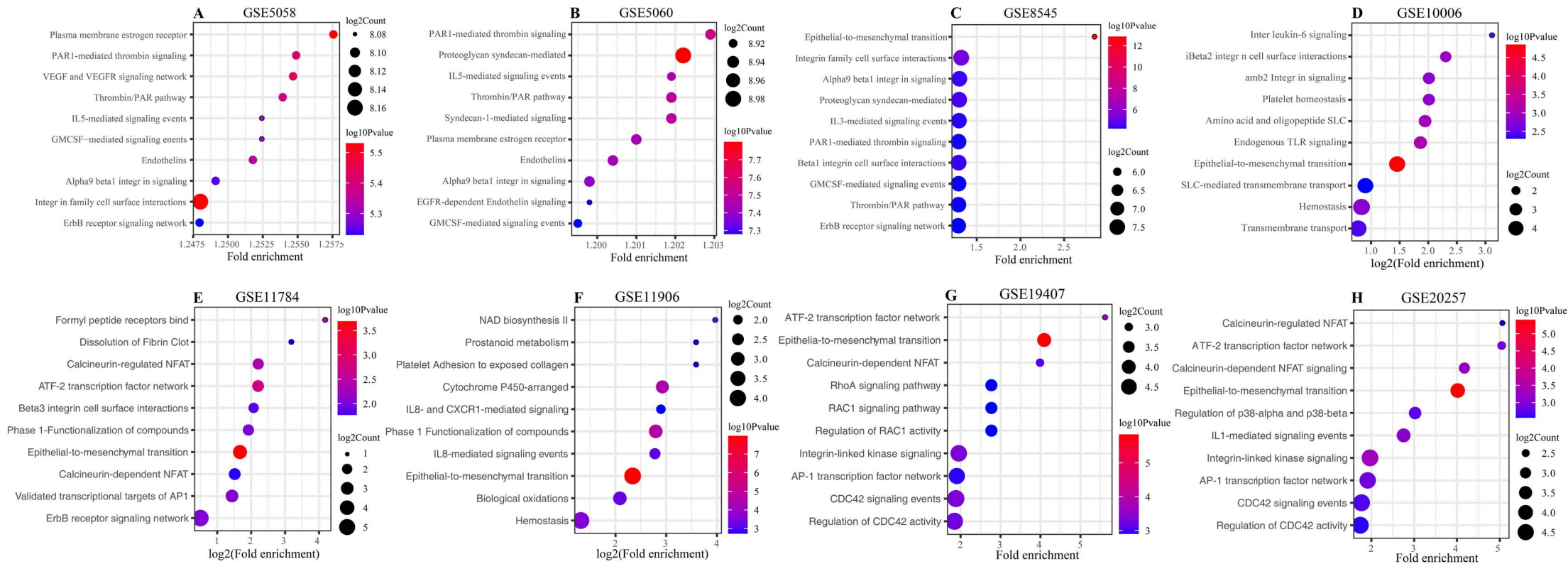

Supplement: Supplementary 1 — Figure S1: Gene Ontology (GO) terms of the 8 separate microarrays in COPD smokers vs. normal smokers. The rectangular length represents counts of the enriched DEGs. The line represents the negative log2P values (MF: molecular function; CC: cellular component; BP: biological process). Figure S2: Gene Ontology (GO) terms of the 8 separate microarrays in COPD smokers vs. normal nonsmokers. The line represents the negative log2P values (MF: molecular function; CC: cellular component; BP: biological process). Figure S3: Gene Ontology (GO) terms of the 8 separate microarrays in normal smokers vs. normal nonsmokers. Figure S4: the Kyoto Encyclopedia of Genes and Genomes (KEGG) pathway enrichment of the 8 separate microarrays in COPD smokers vs. normal smokers. The dot sizes represent counts of the enriched DEGs. The dot colors represent the negative log2P value. Figure S5: the Kyoto Encyclopedia of Genes and Genomes (KEGG) pathway enrichment of the 8 separate microarray data in COPD smokers vs. normal nonsmokers. Figure S6: the Kyoto Encyclopedia of Genes and Genomes (KEGG) pathway enrichment of the 8 separate microarrays in normal smokers vs. normal nonsmokers. Figure S7: the volcano plot of DEGs on the 8 separate microarrays in COPD smokers vs. normal smokers. Blue indicates genes with decreased expression, red indicates genes with increased expression, and white indicates genes with average expression. Figure S8: the volcano plot of DEGs on the 8 separate microarrays in COPD smokers vs. normal nonsmokers. Figure S9: the volcano plot of DEGs on the 8 separate microarrays in normal smokers vs. normal nonsmokers. Figure S10: the expressions of the selected DEG expressions on the 8 separate microarrays. The horizontal axis represents groups, while the vertical axis for DEG expressions. Figure S11: the network between DEMs and differentially expressed miRNAs on the combined 8 human microarray and rat transcriptomic data. (A-C) The network for DEGs in human small airway epith [file 9354286.f1.zip › figures/Figure S4-new.pdf]

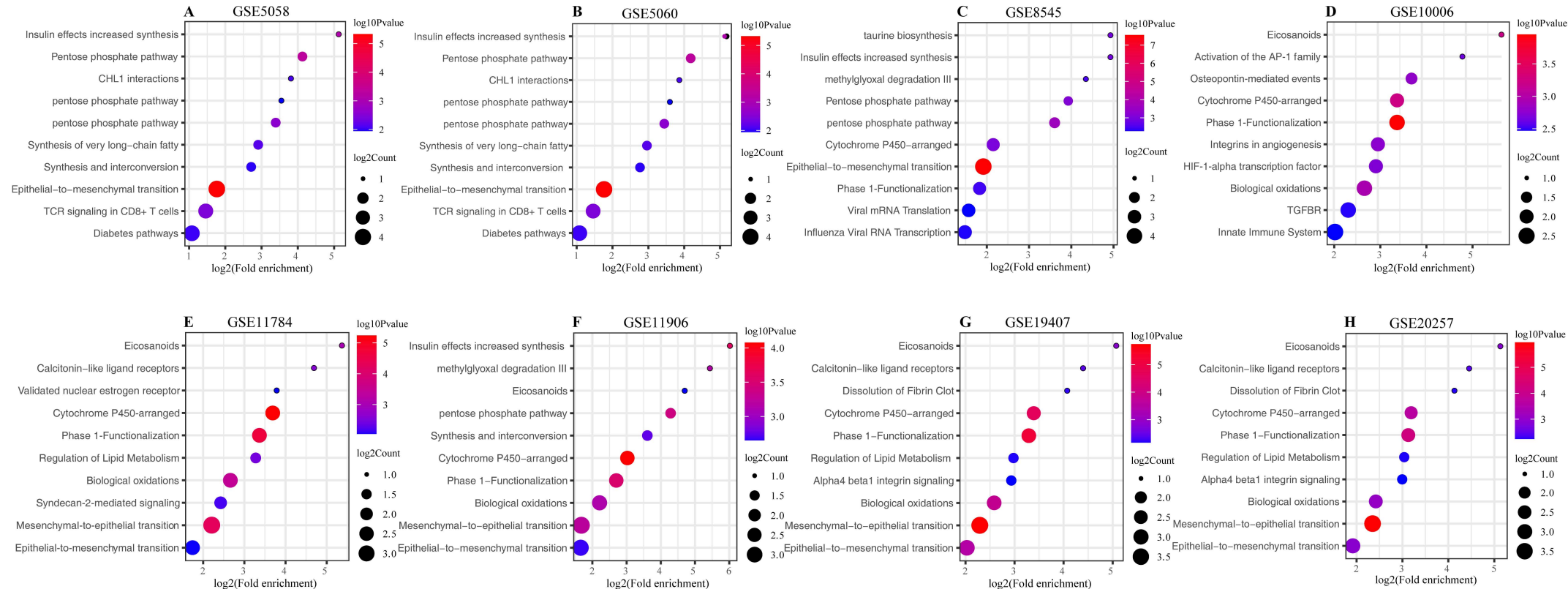

Supplement: Supplementary 1 — Figure S1: Gene Ontology (GO) terms of the 8 separate microarrays in COPD smokers vs. normal smokers. The rectangular length represents counts of the enriched DEGs. The line represents the negative log2P values (MF: molecular function; CC: cellular component; BP: biological process). Figure S2: Gene Ontology (GO) terms of the 8 separate microarrays in COPD smokers vs. normal nonsmokers. The line represents the negative log2P values (MF: molecular function; CC: cellular component; BP: biological process). Figure S3: Gene Ontology (GO) terms of the 8 separate microarrays in normal smokers vs. normal nonsmokers. Figure S4: the Kyoto Encyclopedia of Genes and Genomes (KEGG) pathway enrichment of the 8 separate microarrays in COPD smokers vs. normal smokers. The dot sizes represent counts of the enriched DEGs. The dot colors represent the negative log2P value. Figure S5: the Kyoto Encyclopedia of Genes and Genomes (KEGG) pathway enrichment of the 8 separate microarray data in COPD smokers vs. normal nonsmokers. Figure S6: the Kyoto Encyclopedia of Genes and Genomes (KEGG) pathway enrichment of the 8 separate microarrays in normal smokers vs. normal nonsmokers. Figure S7: the volcano plot of DEGs on the 8 separate microarrays in COPD smokers vs. normal smokers. Blue indicates genes with decreased expression, red indicates genes with increased expression, and white indicates genes with average expression. Figure S8: the volcano plot of DEGs on the 8 separate microarrays in COPD smokers vs. normal nonsmokers. Figure S9: the volcano plot of DEGs on the 8 separate microarrays in normal smokers vs. normal nonsmokers. Figure S10: the expressions of the selected DEG expressions on the 8 separate microarrays. The horizontal axis represents groups, while the vertical axis for DEG expressions. Figure S11: the network between DEMs and differentially expressed miRNAs on the combined 8 human microarray and rat transcriptomic data. (A-C) The network for DEGs in human small airway epith [file 9354286.f1.zip › figures/Figure S5.pdf]

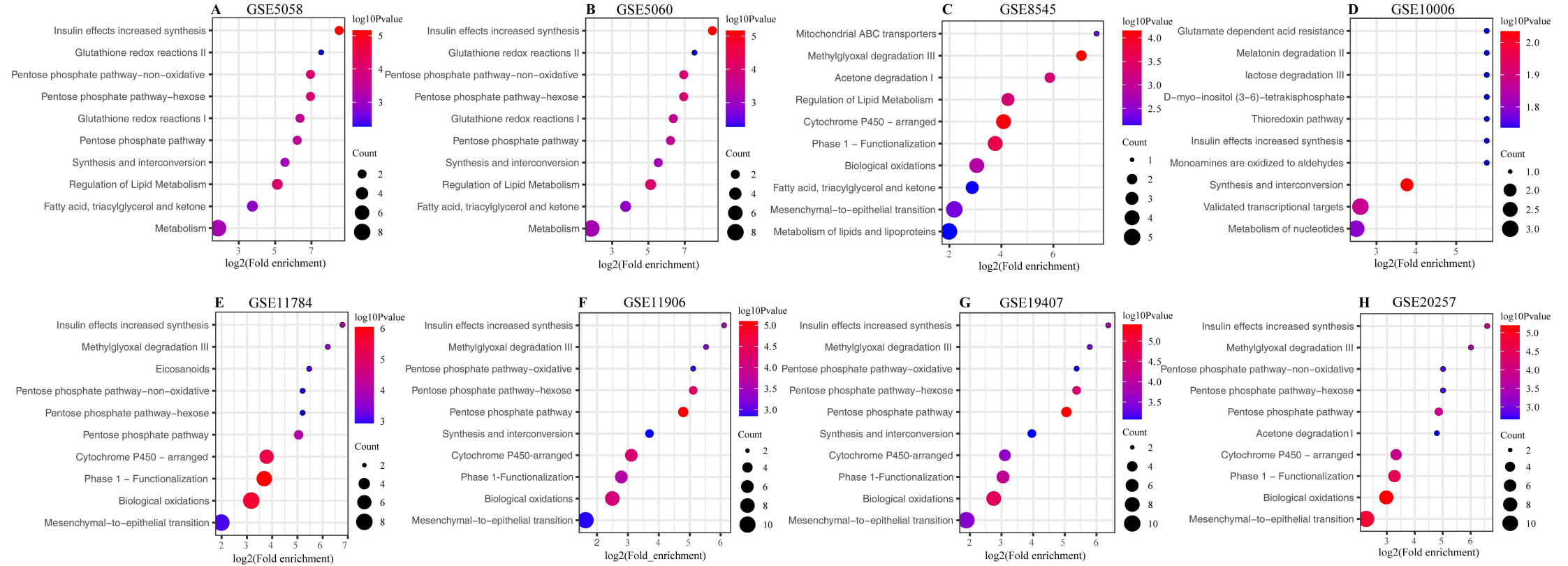

Supplement: Supplementary 1 — Figure S1: Gene Ontology (GO) terms of the 8 separate microarrays in COPD smokers vs. normal smokers. The rectangular length represents counts of the enriched DEGs. The line represents the negative log2P values (MF: molecular function; CC: cellular component; BP: biological process). Figure S2: Gene Ontology (GO) terms of the 8 separate microarrays in COPD smokers vs. normal nonsmokers. The line represents the negative log2P values (MF: molecular function; CC: cellular component; BP: biological process). Figure S3: Gene Ontology (GO) terms of the 8 separate microarrays in normal smokers vs. normal nonsmokers. Figure S4: the Kyoto Encyclopedia of Genes and Genomes (KEGG) pathway enrichment of the 8 separate microarrays in COPD smokers vs. normal smokers. The dot sizes represent counts of the enriched DEGs. The dot colors represent the negative log2P value. Figure S5: the Kyoto Encyclopedia of Genes and Genomes (KEGG) pathway enrichment of the 8 separate microarray data in COPD smokers vs. normal nonsmokers. Figure S6: the Kyoto Encyclopedia of Genes and Genomes (KEGG) pathway enrichment of the 8 separate microarrays in normal smokers vs. normal nonsmokers. Figure S7: the volcano plot of DEGs on the 8 separate microarrays in COPD smokers vs. normal smokers. Blue indicates genes with decreased expression, red indicates genes with increased expression, and white indicates genes with average expression. Figure S8: the volcano plot of DEGs on the 8 separate microarrays in COPD smokers vs. normal nonsmokers. Figure S9: the volcano plot of DEGs on the 8 separate microarrays in normal smokers vs. normal nonsmokers. Figure S10: the expressions of the selected DEG expressions on the 8 separate microarrays. The horizontal axis represents groups, while the vertical axis for DEG expressions. Figure S11: the network between DEMs and differentially expressed miRNAs on the combined 8 human microarray and rat transcriptomic data. (A-C) The network for DEGs in human small airway epith [file 9354286.f1.zip › figures/Figure S6.pdf]

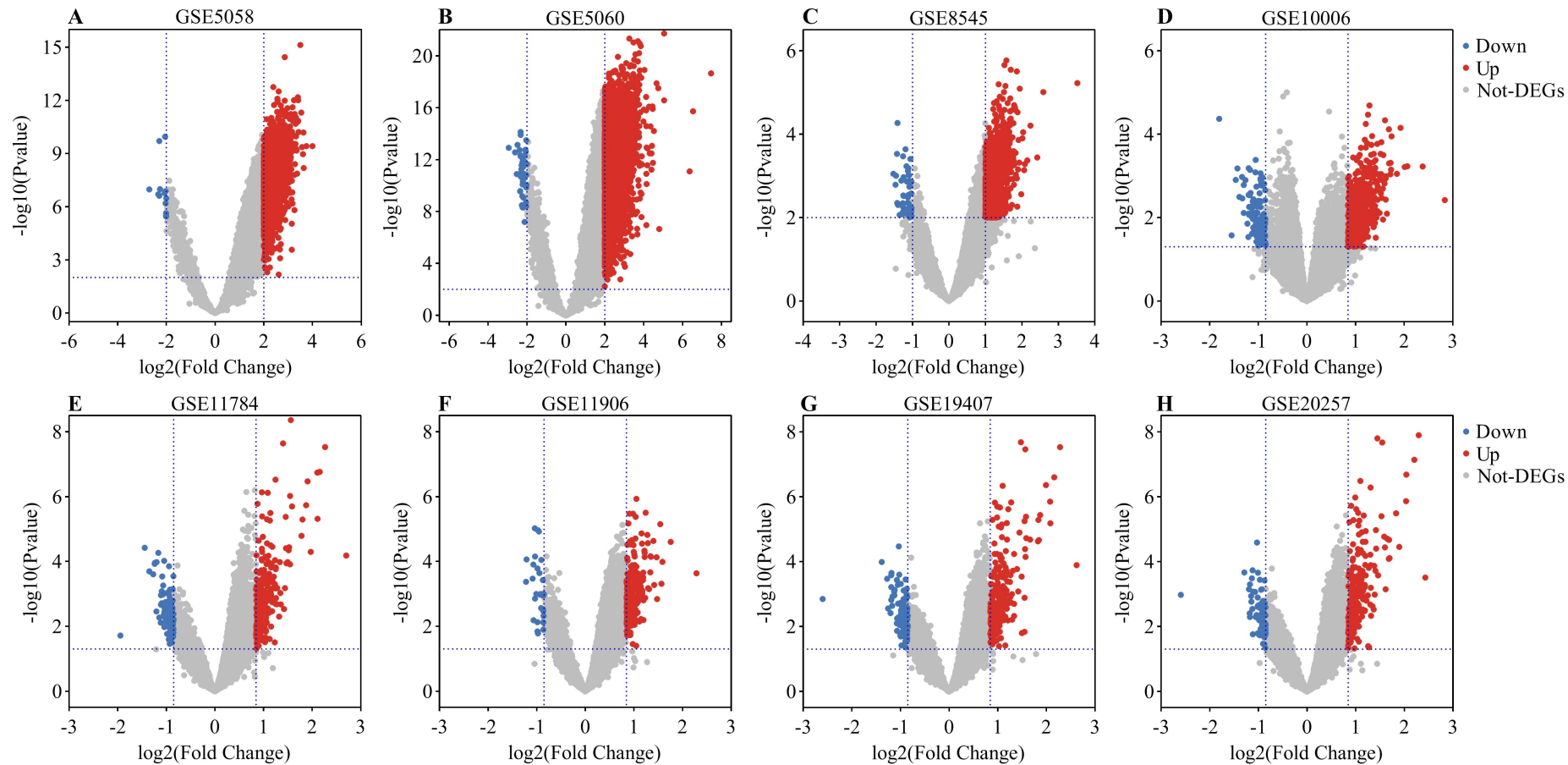

Supplement: Supplementary 1 — Figure S1: Gene Ontology (GO) terms of the 8 separate microarrays in COPD smokers vs. normal smokers. The rectangular length represents counts of the enriched DEGs. The line represents the negative log2P values (MF: molecular function; CC: cellular component; BP: biological process). Figure S2: Gene Ontology (GO) terms of the 8 separate microarrays in COPD smokers vs. normal nonsmokers. The line represents the negative log2P values (MF: molecular function; CC: cellular component; BP: biological process). Figure S3: Gene Ontology (GO) terms of the 8 separate microarrays in normal smokers vs. normal nonsmokers. Figure S4: the Kyoto Encyclopedia of Genes and Genomes (KEGG) pathway enrichment of the 8 separate microarrays in COPD smokers vs. normal smokers. The dot sizes represent counts of the enriched DEGs. The dot colors represent the negative log2P value. Figure S5: the Kyoto Encyclopedia of Genes and Genomes (KEGG) pathway enrichment of the 8 separate microarray data in COPD smokers vs. normal nonsmokers. Figure S6: the Kyoto Encyclopedia of Genes and Genomes (KEGG) pathway enrichment of the 8 separate microarrays in normal smokers vs. normal nonsmokers. Figure S7: the volcano plot of DEGs on the 8 separate microarrays in COPD smokers vs. normal smokers. Blue indicates genes with decreased expression, red indicates genes with increased expression, and white indicates genes with average expression. Figure S8: the volcano plot of DEGs on the 8 separate microarrays in COPD smokers vs. normal nonsmokers. Figure S9: the volcano plot of DEGs on the 8 separate microarrays in normal smokers vs. normal nonsmokers. Figure S10: the expressions of the selected DEG expressions on the 8 separate microarrays. The horizontal axis represents groups, while the vertical axis for DEG expressions. Figure S11: the network between DEMs and differentially expressed miRNAs on the combined 8 human microarray and rat transcriptomic data. (A-C) The network for DEGs in human small airway epith [file 9354286.f1.zip › figures/Figure S7.pdf]

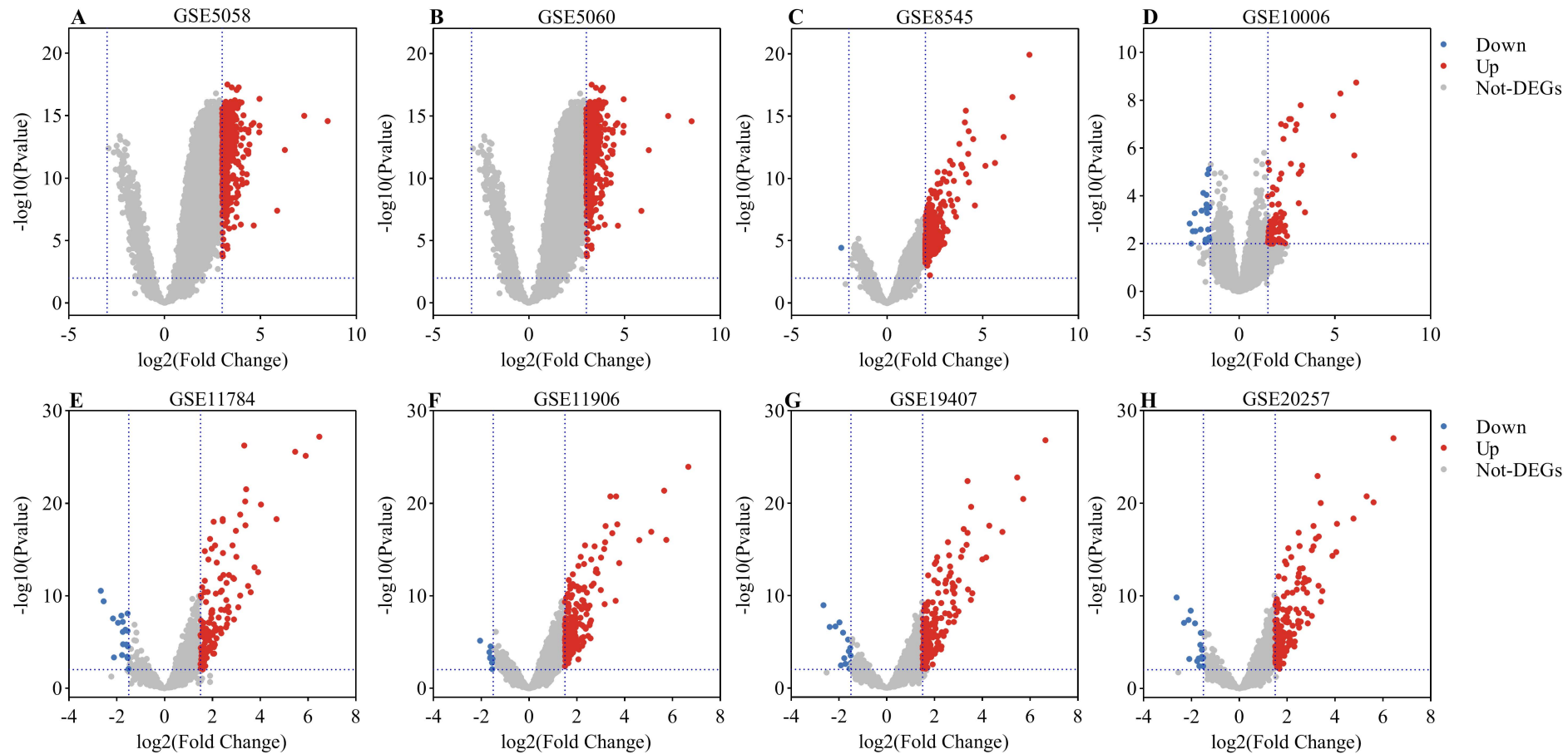

Supplement: Supplementary 1 — Figure S1: Gene Ontology (GO) terms of the 8 separate microarrays in COPD smokers vs. normal smokers. The rectangular length represents counts of the enriched DEGs. The line represents the negative log2P values (MF: molecular function; CC: cellular component; BP: biological process). Figure S2: Gene Ontology (GO) terms of the 8 separate microarrays in COPD smokers vs. normal nonsmokers. The line represents the negative log2P values (MF: molecular function; CC: cellular component; BP: biological process). Figure S3: Gene Ontology (GO) terms of the 8 separate microarrays in normal smokers vs. normal nonsmokers. Figure S4: the Kyoto Encyclopedia of Genes and Genomes (KEGG) pathway enrichment of the 8 separate microarrays in COPD smokers vs. normal smokers. The dot sizes represent counts of the enriched DEGs. The dot colors represent the negative log2P value. Figure S5: the Kyoto Encyclopedia of Genes and Genomes (KEGG) pathway enrichment of the 8 separate microarray data in COPD smokers vs. normal nonsmokers. Figure S6: the Kyoto Encyclopedia of Genes and Genomes (KEGG) pathway enrichment of the 8 separate microarrays in normal smokers vs. normal nonsmokers. Figure S7: the volcano plot of DEGs on the 8 separate microarrays in COPD smokers vs. normal smokers. Blue indicates genes with decreased expression, red indicates genes with increased expression, and white indicates genes with average expression. Figure S8: the volcano plot of DEGs on the 8 separate microarrays in COPD smokers vs. normal nonsmokers. Figure S9: the volcano plot of DEGs on the 8 separate microarrays in normal smokers vs. normal nonsmokers. Figure S10: the expressions of the selected DEG expressions on the 8 separate microarrays. The horizontal axis represents groups, while the vertical axis for DEG expressions. Figure S11: the network between DEMs and differentially expressed miRNAs on the combined 8 human microarray and rat transcriptomic data. (A-C) The network for DEGs in human small airway epith [file 9354286.f1.zip › figures/Figure S8.pdf]

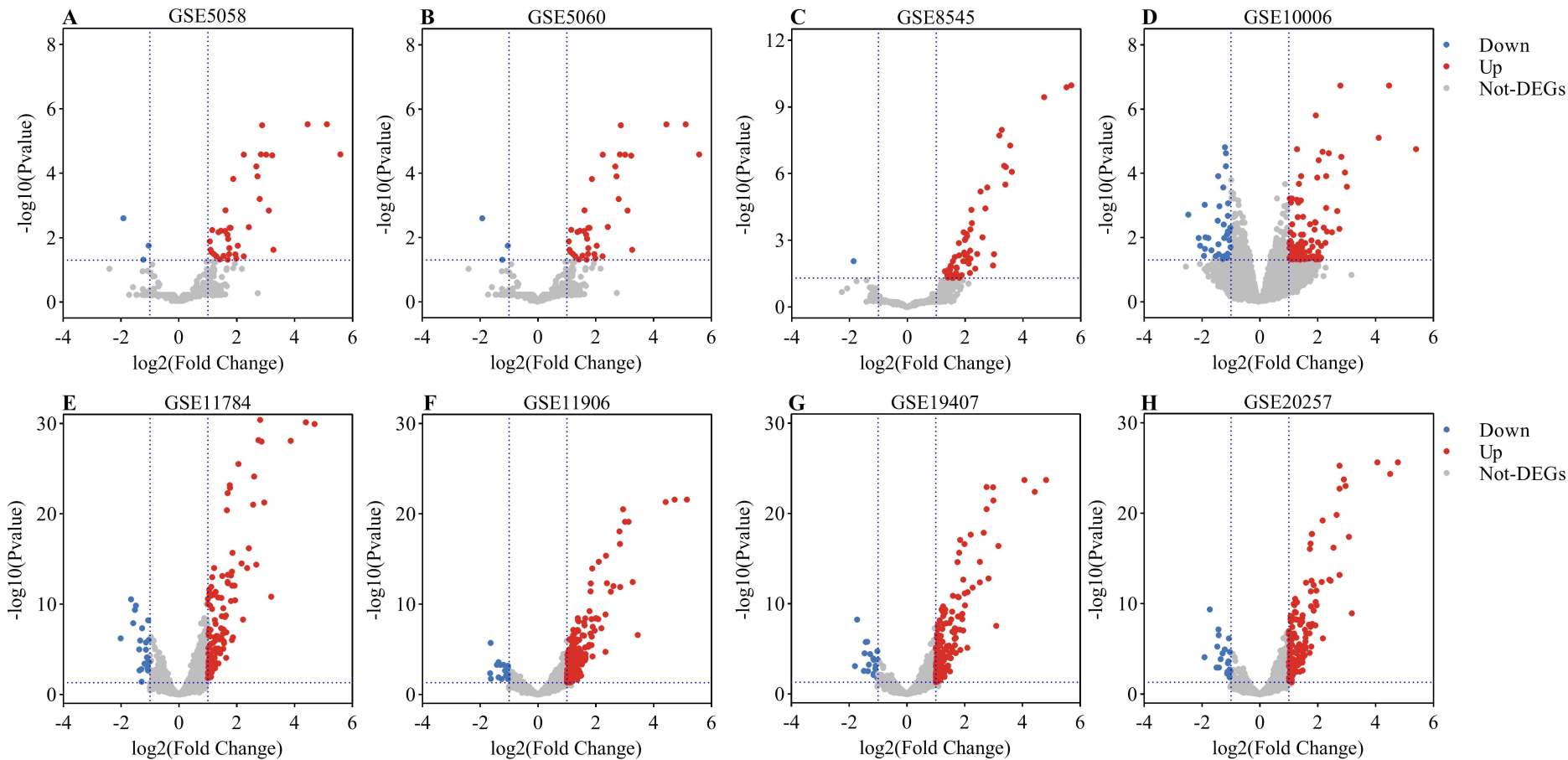

Supplement: Supplementary 1 — Figure S1: Gene Ontology (GO) terms of the 8 separate microarrays in COPD smokers vs. normal smokers. The rectangular length represents counts of the enriched DEGs. The line represents the negative log2P values (MF: molecular function; CC: cellular component; BP: biological process). Figure S2: Gene Ontology (GO) terms of the 8 separate microarrays in COPD smokers vs. normal nonsmokers. The line represents the negative log2P values (MF: molecular function; CC: cellular component; BP: biological process). Figure S3: Gene Ontology (GO) terms of the 8 separate microarrays in normal smokers vs. normal nonsmokers. Figure S4: the Kyoto Encyclopedia of Genes and Genomes (KEGG) pathway enrichment of the 8 separate microarrays in COPD smokers vs. normal smokers. The dot sizes represent counts of the enriched DEGs. The dot colors represent the negative log2P value. Figure S5: the Kyoto Encyclopedia of Genes and Genomes (KEGG) pathway enrichment of the 8 separate microarray data in COPD smokers vs. normal nonsmokers. Figure S6: the Kyoto Encyclopedia of Genes and Genomes (KEGG) pathway enrichment of the 8 separate microarrays in normal smokers vs. normal nonsmokers. Figure S7: the volcano plot of DEGs on the 8 separate microarrays in COPD smokers vs. normal smokers. Blue indicates genes with decreased expression, red indicates genes with increased expression, and white indicates genes with average expression. Figure S8: the volcano plot of DEGs on the 8 separate microarrays in COPD smokers vs. normal nonsmokers. Figure S9: the volcano plot of DEGs on the 8 separate microarrays in normal smokers vs. normal nonsmokers. Figure S10: the expressions of the selected DEG expressions on the 8 separate microarrays. The horizontal axis represents groups, while the vertical axis for DEG expressions. Figure S11: the network between DEMs and differentially expressed miRNAs on the combined 8 human microarray and rat transcriptomic data. (A-C) The network for DEGs in human small airway epith [file 9354286.f1.zip › figures/Figure S9.pdf]
