# Supplementary material for: Gene Expression Trajectories from Normal Nonsmokers to COPD Smokers and Disease Progression Discriminant Modeling in Response to Cigarette Smoking
Source: Dis Markers. 2022 Sep 14;2022:9354286. doi: 10.1155/2022/9354286 (PMC9493146; doi:10.1155/2022/9354286)
Supplement: Supplementary 2 — Table S1: demographic data from 8 combined GEO datasets in GPL570. Table S2: demographic data from 8 single GEO datasets in GPL570. Table S3: detail demographic data from 8 GEO combined datasets. Table S4: demographic data of the validated participants. Table S5: primer sets used for real-time PCR. Table S6: predictive efficacy of single gene. [file 9354286.f2.zip › tables/Table S1.docx]

| **Table S1.** Demographic data from 8 combined GEO datasets in GPL570. | | | | |
| --- | --- | --- | --- | --- |
|  | COPD-smokers | CTL-smokers | CTL-nonsmokers | P value |
| N | 55 | 106 | 78 |  |
| Age | 51.7±7.9 | 43.1±7.6 | 40.8±11.2 | 0.005 |
| ≤60 | 49 | 106 | 72 |  |
| >60 | 6 | 0 | 6 |  |
| Gender |  |  |  | 0.576 |
| Male | 42 | 73 | 54 |  |
| Female | 13 | 33 | 24 |  |
| Smoking |  |  |  | — |
| Yes | 55 | 106 | 0 |  |
| No | 0 | 0 | 78 |  |
| Pack years | 36.9±23.3 | 28.0±16.9 |  | — |
| 0 | 0 | 1 | 78 |  |
| 0-20 | 6 | 33 | 0 |  |
| ≥20 | 49 | 72 | 0 |  |
| Ethnic |  |  |  | 0.038 |
| White | 23 | 25 | 30 |  |
| Black | 26 | 66 | 34 |  |
| Asian | 1 | 0 | 2 |  |
| Hispanic | 5 | 15 | 12 |  |
| GOLD |  |  |  | — |
| Early | 22 | — | — |  |
| I | 15 | — | — |  |
| II | 15 | — | — |  |
| III | 3 | — | — |  |

Data are Mean ± SD, or n.
