# Supplementary material for: Gene Expression Trajectories from Normal Nonsmokers to COPD Smokers and Disease Progression Discriminant Modeling in Response to Cigarette Smoking
Source: Dis Markers. 2022 Sep 14;2022:9354286. doi: 10.1155/2022/9354286 (PMC9493146; doi:10.1155/2022/9354286)
Supplement: Supplementary 2 — Table S1: demographic data from 8 combined GEO datasets in GPL570. Table S2: demographic data from 8 single GEO datasets in GPL570. Table S3: detail demographic data from 8 GEO combined datasets. Table S4: demographic data of the validated participants. Table S5: primer sets used for real-time PCR. Table S6: predictive efficacy of single gene. [file 9354286.f2.zip › tables/Table S2.docx]

| **Table S2.** Demographic data from 8 single GEO datasets in GPL570. | | | | | |
| --- | --- | --- | --- | --- | --- |
| GSE |  | COPD-sm | CTL-sm | CTL-nsm | Pvalue |
| GSE5058 | N | 15 | 12 | 12 |  |
|  | Age | 50.9±6.6 | 46.3±6.4 | 42.3±7.6 | 0.712 |
|  | ≤60 | 14 | 12 | 72 |  |
|  | >60 | 1 | 0 | 6 |  |
|  | Gender |  |  |  | 0.896 |
|  | Male | 11 | 9 | 10 |  |
|  | Female | 4 | 3 | 2 |  |
|  | Smoke |  |  |  | — |
|  | Yes | 15 | 15 | 0 |  |
|  | No | 0 | 0 | 12 |  |
|  | Pack years | 31.5±20.2 | 33.2±19.5 |  | — |
|  | 0 | 0 | 0 | 12 |  |
|  | 0-20 | 3 | 1 | 0 |  |
|  | ≥20 | 12 | 11 | 0 |  |
| GSE5060 | N | 15 | 12 | 12 |  |
|  | Age | 50.9±6.6 | 46.3±6.4 | 42.3±7.6 | 0.712 |
|  | ≤60 | 14 | 12 | 11 |  |
|  | >60 | 1 | 0 | 1 |  |
|  | Gender |  |  |  | 0.896 |
|  | Male | 11 | 9 | 10 |  |
|  | Female | 4 | 3 | 2 |  |
|  | Smoke |  |  |  | — |
|  | Yes | 15 | 12 | 0 |  |
|  | No | 0 | 0 | 15 |  |
|  | Pack years | 31.5±20.2 | 33.2±19.5 |  | — |
|  | 0 | 0 | 0 | 12 |  |
|  | 0-20 | 3 | 1 | 0 |  |
|  | ≥20 | 12 | 11 | 0 |  |
| GSE8545 | N | 18 | 18 | 18 |  |
|  | Age | 49.7±6.4 | 46.2±5.5 | 41.1±7.0 | 1.000 |
|  | ≤60 | 17 | 18 | 17 |  |
|  | >60 | 1 | 0 | 1 |  |
|  | Gender |  |  |  | 0.621 |
|  | Male | 15 | 12 | 14 |  |
|  | Female | 3 | 6 | 4 |  |
|  | Smoke |  |  |  | — |
|  | Yes | 18 | 18 | 0 |  |
|  | No | 0 | 0 | 18 |  |
|  | Pack years | 36.9±23.3 | 28.0±16.9 |  | — |
|  | 0 | 0 | 0 | 18 |  |
|  | 0-20 | 3 | 3 | 0 |  |
|  | ≥20 | 15 | 15 |  |  |
| GSE20257 | N | 23 | 59 | 53 |  |
|  | Age | 51.8±8.4 | 43.0±7.2 | 41.2±11.3 | 0.012 |
|  | ≤60 | 20 | 59 | 49 |  |
|  | >60 | 3 | 0 | 4 |  |
|  | Gender |  |  |  | 0.484 |
|  | Male | 18 | 39 | 39 |  |
|  | Female | 5 | 20 | 14 |  |
|  | Smoke |  |  |  | — |
|  | Yes | 23 | 59 | 0 |  |
|  | No | 0 | 0 | 53 |  |
|  | Pack years | 40.8±27.5 | 28.3±16.8 |  | — |
|  | 0 | 0 | 0 | 53 |  |
|  | 0-20 | 2 | 17 | 0 |  |
|  | ≥20 | 21 | 42 | 0 |  |
| GSE19407 | N | 22 | 58 | 47 |  |
|  | Age | 51.5±8.5 | 42.9±7.2 | 42.1±11.4 | 0.012 |
|  | ≤60 | 19 | 58 | 43 |  |
|  | >60 | 3 | 0 | 4 |  |
|  | Gender |  |  |  | 0.344 |
|  | Male | 18 | 38 | 34 |  |
|  | Female | 4 | 20 | 13 |  |
|  | Smoke |  |  |  | — |
|  | Yes | 22 | 58 | 0 |  |
|  | No | 0 | 0 | 47 |  |
|  | Pack years | 40.9±28.2 | 27.9±16.7 |  | — |
|  | 0 | 0 | 0 | 47 |  |
|  | 0-20 | 2 | 17 | 0 |  |
|  | ≥20 | 20 | 41 | 0 |  |
| GSE11906 | N | 33 | 63 | 35 |  |
|  | Age | 51.5±7.7 | 43.5±6.9 | 43.4±10.3 | 0.023 |
|  | ≤60 | 30 | 63 | 32 |  |
|  | >60 | 3 | 0 | 3 |  |
|  | Gender |  |  |  | 0.287 |
|  | Male | 26 | 41 | 27 |  |
|  | Female | 7 | 22 | 8 |  |
|  | Smoke |  |  |  | — |
|  | Yes | 33 | 63 | 0 |  |
|  | No | 0 | 0 | 35 |  |
|  | Pack years | 36.4±21.9 | 27.4±15.7 |  | — |
|  | 0 | 0 | 0 | 35 |  |
|  | 0-20 | 4 | 19 | 0 |  |
|  | ≥20 | 29 | 44 | 0 |  |
| GSE11784 | N | 22 | 72 | 63 |  |
|  | Age | 51.5±8.5 | 42.4±7.6 | 40.5±12.0 | 0.005 |
|  | ≤60 | 19 | 72 | 58 |  |
|  | >60 | 3 | 0 | 5 |  |
|  | Gender |  |  |  | 0.308 |
|  | Male | 18 | 52 | 41 |  |
|  | Female | 4 | 20 | 22 |  |
|  | Smoke |  |  |  | — |
|  | Yes | 22 | 72 | 0 |  |
|  | No | 0 | 0 | 63 |  |
|  | Pack years | 40.9±28.2 | 27.2±15.9 |  | — |
|  | 0 | 0 | 0 | 63 |  |
|  | 0-20 | 2 | 23 | 0 |  |
|  | ≥20 | 20 | 49 | 0 |  |
| GSE10006 | N | 27 | 18 | 13 |  |
|  | Age | 52.4±73.4 | 45.6±4.9 | 42.7±7.2 | 0.590 |
|  | ≤60 | 25 | 18 | 12 |  |
|  | >60 | 2 | 0 | 1 |  |
|  | Gender |  |  |  | 0.902 |
|  | Male | 42 | 73 | 54 |  |
|  | Female | 13 | 33 | 24 |  |
|  | Smoke |  |  |  | — |
|  | Yes | 27 | 18 | 0 |  |
|  | No | 0 | 0 | 13 |  |
|  | Pack years | 39.3±23.1 | 34.6±21.1 | — | — |
|  | 0 |  | 1 | 13 |  |
|  | 0-20 | 1 | 2 | 0 |  |
|  | ≥20 | 26 | 15 | 0 |  |

Data are mean ± SD, or n.
