# Supplementary material for: Gene Expression Trajectories from Normal Nonsmokers to COPD Smokers and Disease Progression Discriminant Modeling in Response to Cigarette Smoking
Source: Dis Markers. 2022 Sep 14;2022:9354286. doi: 10.1155/2022/9354286 (PMC9493146; doi:10.1155/2022/9354286)
Supplement: Supplementary 2 — Table S1: demographic data from 8 combined GEO datasets in GPL570. Table S2: demographic data from 8 single GEO datasets in GPL570. Table S3: detail demographic data from 8 GEO combined datasets. Table S4: demographic data of the validated participants. Table S5: primer sets used for real-time PCR. Table S6: predictive efficacy of single gene. [file 9354286.f2.zip › tables/Table S3.docx]

| **Table S3**. Detail demographic data from 8 GEO combined datasets. | | | | | | | | | |
| --- | --- | --- | --- | --- | --- | --- | --- | --- | --- |
| GSE | sample | group^a^ | COPD | age | sex^b^ | ethnic^c^ | pys^d^ | smoking | GOLD^e^ |
| GSE8545 | GSM190151 | 3 | 0 | 44 | 1 | 2 | 0 | 0 |  |
| GSE8545 | GSM190153 | 3 | 0 | 45 | 2 | 4 | 0 | 0 |  |
| GSE8545 | GSM252830 | 1 | 1 | 50 | 1 | 1 | 35 | 1 | 2 |
| GSE8545 | GSM252833 | 1 | 1 | 59 | 1 | 1 | 48 | 1 | 1 |
| GSE8545 | GSM252835 | 1 | 1 | 51 | 1 | 2 | 75 | 1 | 2 |
| GSE8545 | GSM252836 | 1 | 1 | 46 | 1 | 2 | 27 | 1 | 2 |
| GSE8545 | GSM252837 | 1 | 1 | 56 | 1 | 1 | 60 | 1 | 2 |
| GSE8545 | GSM252839 | 1 | 1 | 46 | 1 | 4 | 22 | 1 | 1 |
| GSE8545 | GSM252841 | 1 | 1 | 52 | 1 | 4 | 23 | 1 | 1 |
| GSE8545 | GSM252867 | 3 | 0 | 45 | 1 | 1 | 0 | 0 |  |
| GSE8545 | GSM252879 | 2 | 0 | 41 | 1 | 2 | 20 | 1 |  |
| GSE8545 | GSM252882 | 2 | 0 | 48 | 1 | 2 | 32 | 1 |  |
| GSE8545 | GSM252884 | 2 | 0 | 43 | 2 | 2 | 36 | 1 |  |
| GSE8545 | GSM252885 | 2 | 0 | 41 | 1 | 2 | 15 | 1 |  |
| GSE8545 | GSM254149 | 3 | 0 | 41 | 2 | 2 | 0 | 0 |  |
| GSE8545 | GSM254150 | 3 | 0 | 35 | 1 | 2 | 0 | 0 |  |
| GSE8545 | GSM254151 | 3 | 0 | 37 | 1 | 2 | 0 | 0 |  |
| GSE8545 | GSM254152 | 3 | 0 | 31 | 1 | 1 | 0 | 0 |  |
| GSE8545 | GSM254157 | 2 | 0 | 45 | 1 | 1 | 23 | 1 |  |
| GSE8545 | GSM254158 | 2 | 0 | 50 | 2 | 2 | 22 | 1 |  |
| GSE8545 | GSM254159 | 2 | 0 | 46 | 2 | 1 | 33 | 1 |  |
| GSE8545 | GSM254160 | 2 | 0 | 49 | 1 | 1 | 16 | 1 |  |
| GSE8545 | GSM254161 | 2 | 0 | 40 | 2 | 2 | 47 | 1 |  |
| GSE8545 | GSM254163 | 1 | 1 | 51 | 2 | 4 | 27.5 | 1 | 2 |
| GSE8545 | GSM254169 | 1 | 1 | 48 | 2 | 1 | 34 | 1 | 2 |
| GSE8545 | GSM254171 | 1 | 1 | 44 | 1 | 2 | 57 | 1 | 1 |
| GSE8545 | GSM254172 | 1 | 1 | 53 | 2 | 2 | 15 | 1 | 2 |
| GSE8545 | GSM254173 | 1 | 1 | 42 | 1 | 2 | 29 | 1 | 2 |
| GSE8545 | GSM254174 | 1 | 1 | 36 | 1 | 4 | 32.5 | 1 | 1 |
| GSE8545 | GSM254175 | 1 | 1 | 44 | 1 | 2 | 14 | 1 | 1 |
| GSE8545 | GSM254176 | 1 | 1 | 62 | 1 | 1 | 24 | 1 | 1 |
| GSE5058 | GSM101095 | 3 | 0 | 41 | 1 | 2 | 0 | 0 |  |
| GSE5058 | GSM101096 | 3 | 0 | 35 | 1 | 2 | 0 | 0 |  |
| GSE5058 | GSM101097 | 3 | 0 | 61 | 1 | 1 | 0 | 0 |  |
| GSE5058 | GSM101098 | 3 | 0 | 37 | 2 | 2 | 0 | 0 |  |
| GSE5058 | GSM101099 | 3 | 0 | 45 | 1 | 4 | 0 | 0 |  |
| GSE5058 | GSM101100 | 3 | 0 | 47 | 1 | 2 | 0 | 0 |  |
| GSE5058 | GSM101101 | 3 | 0 | 38 | 1 | 4 | 0 | 0 |  |
| GSE5058 | GSM101102 | 3 | 0 | 49 | 2 | 1 | 0 | 0 |  |
| GSE5058 | GSM101103 | 3 | 0 | 45 | 1 | 1 | 0 | 0 |  |
| GSE5058 | GSM101104 | 3 | 0 | 36 | 1 | 1 | 0 | 0 |  |
| GSE5058 | GSM101105 | 3 | 0 | 38 | 1 | 2 | 0 | 0 |  |
| GSE5058 | GSM101106 | 3 | 0 | 35 | 1 | 2 | 0 | 0 |  |
| GSE5058 | GSM101107 | 2 | 0 | 46 | 1 | 1 | 21 | 1 |  |
| GSE5058 | GSM101108 | 2 | 0 | 40 | 2 | 2 | 25 | 1 |  |
| GSE5058 | GSM101109 | 2 | 0 | 44 | 1 | 1 | 45 | 1 |  |
| GSE5058 | GSM101110 | 2 | 0 | 43 | 1 | 1 | 15 | 1 |  |
| GSE5058 | GSM101111 | 2 | 0 | 37 | 2 | 2 | 23 | 1 |  |
| GSE5058 | GSM101112 | 2 | 0 | 41 | 1 | 2 | 20 | 1 |  |
| GSE5058 | GSM101113 | 2 | 0 | 45 | 1 | 2 | 28 | 1 |  |
| GSE5058 | GSM101114 | 2 | 0 | 48 | 1 | 1 | 20 | 1 |  |
| GSE5058 | GSM101115 | 2 | 0 | 50 | 1 | 1 | 38 | 1 |  |
| GSE5058 | GSM101116 | 2 | 0 | 46 | 2 | 2 | 23 | 1 |  |
| GSE5058 | GSM114089 | 2 | 0 | 56 | 1 | 2 | 80 | 1 |  |
| GSE5058 | GSM114090 | 2 | 0 | 59 | 1 | 2 | 60 | 1 |  |
| GSE5058 | GSM114091 | 1 | 1 | 39 | 1 | 2 | 13 | 1 | 1 |
| GSE5058 | GSM114092 | 1 | 1 | 47 | 1 | 1 | 50 | 1 | 1 |
| GSE5058 | GSM114093 | 1 | 1 | 47 | 1 | 2 | 33 | 1 | 2 |
| GSE5058 | GSM114094 | 1 | 1 | 52 | 1 | 1 | 18 | 1 | 2 |
| GSE5058 | GSM114095 | 1 | 1 | 57 | 1 | 1 | 20 | 1 | 1 |
| GSE5058 | GSM114096 | 1 | 1 | 54 | 2 | 1 | 20 | 1 | 1 |
| GSE5058 | GSM114097 | 1 | 1 | 48 | 1 | 2 | 23 | 1 | 0 |
| GSE5058 | GSM114098 | 1 | 1 | 61 | 1 | 2 | 20 | 1 | 0 |
| GSE5058 | GSM114099 | 1 | 1 | 48 | 1 | 2 | 12 | 1 | 0 |
| GSE5058 | GSM114100 | 1 | 1 | 59 | 2 | 2 | 30 | 1 | 0 |
| GSE5058 | GSM114101 | 1 | 1 | 45 | 2 | 1 | 43 | 1 | 0 |
| GSE5058 | GSM114102 | 1 | 1 | 55 | 2 | 1 | 31 | 1 | 0 |
| GSE5058 | GSM114103 | 1 | 1 | 41 | 1 | 1 | 50 | 1 | 0 |
| GSE5058 | GSM114104 | 1 | 1 | 52 | 1 | 4 | 90 | 1 | 0 |
| GSE5058 | GSM114105 | 1 | 1 | 59 | 1 | 2 | 20 | 1 | 0 |
| GSE20257 | GSM252829 | 1 | 1 | 47 | 1 | 2 | 33 | 1 | 2 |
| GSE20257 | GSM364037 | 1 | 1 | 57 | 2 | 1 | 38.5 | 1 | 2 |
| GSE11906 | GSM190152 | 3 | 0 | 45 | 1 | 1 | 0 | 0 |  |
| GSE11906 | GSM252834 | 1 | 1 | 56 | 1 | 1 | 35 | 1 | 1 |
| GSE11906 | GSM252840 | 1 | 1 | 73 | 2 | 2 | 53 | 1 | 3 |
| GSE11906 | GSM252842 | 1 | 1 | 48 | 1 | 2 | 23 | 1 | 0 |
| GSE11906 | GSM252843 | 1 | 1 | 61 | 1 | 2 | 20 | 1 | 0 |
| GSE11906 | GSM252844 | 1 | 1 | 48 | 1 | 2 | 12 | 1 | 0 |
| GSE11906 | GSM252845 | 1 | 1 | 59 | 2 | 2 | 30 | 1 | 0 |
| GSE11906 | GSM252846 | 1 | 1 | 45 | 2 | 1 | 42.5 | 1 | 0 |
| GSE11906 | GSM252847 | 1 | 1 | 55 | 2 | 1 | 31 | 1 | 0 |
| GSE11906 | GSM252848 | 1 | 1 | 42 | 1 | 2 | 30 | 1 | 0 |
| GSE11906 | GSM252849 | 1 | 1 | 41 | 1 | 1 | 50 | 1 | 0 |
| GSE11906 | GSM252850 | 1 | 1 | 52 | 1 | 2 | 90 | 1 | 0 |
| GSE11906 | GSM252851 | 1 | 1 | 59 | 1 | 2 | 20 | 1 | 0 |
| GSE11906 | GSM252852 | 1 | 1 | 59 | 1 | 1 | 50 | 1 | 0 |
| GSE11906 | GSM252853 | 1 | 1 | 50 | 1 | 2 | 32 | 1 | 0 |
| GSE11906 | GSM252854 | 1 | 1 | 41 | 1 | 2 | 20 | 1 | 0 |
| GSE11906 | GSM252878 | 2 | 0 | 45 | 1 | 2 | 70 | 1 |  |
| GSE11906 | GSM300860 | 3 | 0 | 35 | 1 | 1 | 0 | 0 |  |
| GSE11906 | GSM300861 | 2 | 0 | 38 | 1 | 2 | 38 | 1 |  |
| GSE11906 | GSM300862 | 2 | 0 | 51 | 2 | 2 | 26 | 1 |  |
| GSE11906 | GSM300863 | 2 | 0 | 47 | 1 | 2 | 18 | 1 |  |
| GSE11906 | GSM300864 | 2 | 0 | 43 | 1 | 2 | 7 | 1 |  |
| GSE11906 | GSM300865 | 2 | 0 | 42 | 1 | 2 | 15 | 1 |  |
| GSE11906 | GSM300866 | 2 | 0 | 46 | 1 | 2 | 49 | 1 |  |
| GSE11906 | GSM300867 | 2 | 0 | 37 | 2 | 2 | 30 | 1 |  |
| GSE11906 | GSM300868 | 2 | 0 | 52 | 2 | 2 | 35 | 1 |  |
| GSE11906 | GSM300869 | 2 | 0 | 44 | 2 | 2 | 11 | 1 |  |
| GSE11906 | GSM300870 | 2 | 0 | 59 | 1 | 2 | 40 | 1 |  |
| GSE11906 | GSM300871 | 2 | 0 | 43 | 1 | 4 | 10 | 1 |  |
| GSE11906 | GSM300872 | 2 | 0 | 36 | 1 | 2 | 23 | 1 |  |
| GSE11906 | GSM300873 | 2 | 0 | 42 | 2 | 4 | 29 | 1 |  |
| GSE11906 | GSM300874 | 2 | 0 | 31 | 1 | 4 | 13 | 1 |  |
| GSE11906 | GSM300875 | 2 | 0 | 39 | 1 | 2 | 13 | 1 |  |
| GSE11906 | GSM300876 | 2 | 0 | 54 | 2 | 1 | 20 | 1 |  |
| GSE11906 | GSM300877 | 2 | 0 | 42 | 2 | 1 | 28 | 1 |  |
| GSE11906 | GSM300878 | 2 | 0 | 55 | 2 | 1 | 35 | 1 |  |
| GSE11906 | GSM300879 | 2 | 0 | 44 | 1 | 2 | 57 | 1 |  |
| GSE11906 | GSM300880 | 2 | 0 | 20 | 2 | 2 | 8 | 1 |  |
| GSE11784 | GSM190149 | 3 | 0 | 49 | 1 | 1 | 0 | 0 |  |
| GSE11784 | GSM190150 | 3 | 0 | 34 | 1 | 2 | 0 | 0 |  |
| GSE11784 | GSM190154 | 3 | 0 | 29 | 2 | 2 | 0 | 0 |  |
| GSE11784 | GSM190155 | 3 | 0 | 42 | 1 | 2 | 0 | 0 |  |
| GSE11784 | GSM190156 | 3 | 0 | 56 | 1 | 1 | 0 | 0 |  |
| GSE11784 | GSM252828 | 1 | 1 | 47 | 1 | 1 | 50 | 1 | 1 |
| GSE11784 | GSM252831 | 1 | 1 | 55 | 1 | 1 | 20 | 1 | 2 |
| GSE11784 | GSM252838 | 1 | 1 | 60 | 1 | 3 | 110 | 1 | 3 |
| GSE11784 | GSM252855 | 3 | 0 | 41 | 1 | 2 | 0 | 0 |  |
| GSE11784 | GSM252871 | 2 | 0 | 40 | 1 | 2 | 24 | 1 |  |
| GSE11784 | GSM252876 | 2 | 0 | 45 | 1 | 2 | 24 | 1 |  |
| GSE11784 | GSM252880 | 2 | 0 | 47 | 1 | 2 | 29 | 1 |  |
| GSE11784 | GSM252881 | 2 | 0 | 41 | 1 | 2 | 45 | 1 |  |
| GSE11784 | GSM298219 | 3 | 0 | 44 | 1 | 1 | 0 | 0 |  |
| GSE11784 | GSM298220 | 3 | 0 | 60 | 1 | 1 | 0 | 0 |  |
| GSE11784 | GSM298221 | 3 | 0 | 49 | 1 | 1 | 0 | 0 |  |
| GSE11784 | GSM298222 | 3 | 0 | 49 | 1 | 1 | 0 | 0 |  |
| GSE11784 | GSM298223 | 3 | 0 | 38 | 1 | 4 | 0 | 0 |  |
| GSE11784 | GSM298224 | 3 | 0 | 73 | 1 | 1 | 0 | 0 |  |
| GSE11784 | GSM298225 | 3 | 0 | 49 | 1 | 2 | 0 | 0 |  |
| GSE11784 | GSM298226 | 3 | 0 | 22 | 2 | 2 | 0 | 0 |  |
| GSE11784 | GSM298227 | 3 | 0 | 29 | 1 | 4 | 0 | 0 |  |
| GSE11784 | GSM298228 | 3 | 0 | 39 | 2 | 3 | 0 | 0 |  |
| GSE11784 | GSM298229 | 3 | 0 | 48 | 2 | 2 | 0 | 0 |  |
| GSE11784 | GSM298230 | 2 | 0 | 39 | 1 | 4 | 30 | 1 |  |
| GSE11784 | GSM298231 | 2 | 0 | 54 | 2 | 1 | 45 | 1 |  |
| GSE11784 | GSM298232 | 2 | 0 | 43 | 1 | 2 | 30 | 1 |  |
| GSE11784 | GSM298233 | 2 | 0 | 36 | 1 | 4 | 3 | 1 |  |
| GSE11784 | GSM298234 | 2 | 0 | 41 | 2 | 2 | 22.5 | 1 |  |
| GSE11784 | GSM298235 | 2 | 0 | 46 | 2 | 2 | 19 | 1 |  |
| GSE11784 | GSM298236 | 2 | 0 | 47 | 1 | 2 | 11 | 1 |  |
| GSE11784 | GSM298237 | 2 | 0 | 41 | 1 | 1 | 12 | 1 |  |
| GSE11784 | GSM298238 | 2 | 0 | 42 | 2 | 1 | 20 | 1 |  |
| GSE11784 | GSM298239 | 2 | 0 | 46 | 1 | 4 | 26 | 1 |  |
| GSE11784 | GSM298240 | 2 | 0 | 41 | 1 | 4 | 13 | 1 |  |
| GSE11784 | GSM298241 | 2 | 0 | 32 | 2 | 2 | 7.6 | 1 |  |
| GSE11784 | GSM298242 | 2 | 0 | 27 | 2 | 2 | 3.8 | 1 |  |
| GSE11784 | GSM298243 | 2 | 0 | 35 | 1 | 2 | 5 | 1 |  |
| GSE11784 | GSM298244 | 2 | 0 | 40 | 1 | 2 | 44.3 | 1 |  |
| GSE11784 | GSM298245 | 2 | 0 | 48 | 1 | 2 | 43 | 1 |  |
| GSE11784 | GSM298246 | 2 | 0 | 47 | 1 | 2 | 33 | 1 |  |
| GSE11784 | GSM298247 | 2 | 0 | 41 | 1 | 2 | 38 | 1 |  |
| GSE11784 | GSM300859 | 3 | 0 | 62 | 2 | 1 | 0 | 0 |  |
| GSE11784 | GSM302396 | 3 | 0 | 47 | 1 | 1 | 0 | 0 |  |
| GSE11784 | GSM302397 | 3 | 0 | 39 | 1 | 2 | 0 | 0 |  |
| GSE11784 | GSM302399 | 2 | 0 | 27 | 2 | 1 | 38 | 1 |  |
| GSE11784 | GSM350871 | 3 | 0 | 24 | 1 | 2 | 0 | 0 |  |
| GSE11784 | GSM350873 | 3 | 0 | 31 | 1 | 2 | 0 | 0 |  |
| GSE11784 | GSM350874 | 2 | 0 | 43 | 2 | 2 | 17.5 | 1 |  |
| GSE11784 | GSM350955 | 3 | 0 | 26 | 1 | 2 | 0 | 0 |  |
| GSE11784 | GSM350956 | 3 | 0 | 33 | 2 | 1 | 0 | 0 |  |
| GSE11784 | GSM350957 | 2 | 0 | 45 | 1 | 2 | 46 | 1 |  |
| GSE11784 | GSM350958 | 2 | 0 | 48 | 2 | 2 | 26.5 | 1 |  |
| GSE11784 | GSM364038 | 1 | 1 | 66 | 1 | 1 | 119 | 1 | 1 |
| GSE11784 | GSM364041 | 1 | 1 | 45 | 1 | 1 | 26 | 1 | 1 |
| GSE11784 | GSM364045 | 1 | 1 | 45 | 1 | 2 | 24 | 1 | 2 |
| GSE11784 | GSM364046 | 2 | 0 | 48 | 2 | 2 | 0.5 | 1 |  |
| GSE11784 | GSM364048 | 2 | 0 | 47 | 2 | 2 | 56.5 | 1 |  |
| GSE11784 | GSM410161 | 3 | 0 | 21 | 2 | 2 | 0 | 0 |  |
| GSE11784 | GSM410162 | 3 | 0 | 45 | 1 | 2 | 0 | 0 |  |
| GSE11784 | GSM410163 | 3 | 0 | 55 | 1 | 2 | 0 | 0 |  |
| GSE11784 | GSM410164 | 2 | 0 | 47 | 1 | 2 | 45 | 1 |  |
| GSE11784 | GSM410165 | 2 | 0 | 39 | 1 | 2 | 11 | 1 |  |
| GSE11784 | GSM434049 | 3 | 0 | 68 | 1 | 1 | 0 | 0 |  |
| GSE11784 | GSM434050 | 3 | 0 | 26 | 2 | 4 | 0 | 0 |  |
| GSE11784 | GSM434051 | 3 | 0 | 45 | 2 | 2 | 0 | 0 |  |
| GSE11784 | GSM434052 | 3 | 0 | 40 | 1 | 1 | 0 | 0 |  |
| GSE11784 | GSM434053 | 2 | 0 | 40 | 1 | 4 | 29 | 1 |  |
| GSE11784 | GSM434054 | 2 | 0 | 46 | 1 | 1 | 47 | 1 |  |
| GSE11784 | GSM434055 | 2 | 0 | 47 | 1 | 2 | 19.5 | 1 |  |
| GSE11784 | GSM434056 | 2 | 0 | 29 | 1 | 4 | 27 | 1 |  |
| GSE11784 | GSM434057 | 2 | 0 | 30 | 1 | 4 | 10 | 1 |  |
| GSE11784 | GSM434058 | 2 | 0 | 47 | 1 | 1 | 24 | 1 |  |
| GSE11784 | GSM434059 | 2 | 0 | 43 | 2 | 4 | 71 | 1 |  |
| GSE11784 | GSM434060 | 2 | 0 | 48 | 1 | 1 | 46 | 1 |  |
| GSE11784 | GSM434061 | 2 | 0 | 24 | 2 | 4 | 10.5 | 1 |  |
| GSE11784 | GSM434062 | 2 | 0 | 27 | 2 | 2 | 1 | 1 |  |
| GSE11784 | GSM434063 | 2 | 0 | 54 | 1 | 2 | 26 | 1 |  |
| GSE11784 | GSM434064 | 1 | 1 | 73 | 2 | 2 | 53 | 1 | 3 |
| GSE11784 | GSM458579 | 3 | 0 | 27 | 1 | 1 | 0 | 0 |  |
| GSE11784 | GSM458580 | 3 | 0 | 34 | 1 | 4 | 0 | 0 |  |
| GSE11784 | GSM458581 | 3 | 0 | 27 | 1 | 2 | 0 | 0 |  |
| GSE11784 | GSM458582 | 3 | 0 | 47 | 2 | 1 | 0 | 0 |  |
| GSE11784 | GSM469989 | 3 | 0 | 55 | 2 | 2 | 0 | 0 |  |
| GSE11784 | GSM469990 | 3 | 0 | 24 | 2 | 4 | 0 | 0 |  |
| GSE11784 | GSM469991 | 3 | 0 | 37 | 1 | 4 | 0 | 0 |  |
| GSE11784 | GSM469992 | 3 | 0 | 44 | 1 | 4 | 0 | 0 |  |
| GSE11784 | GSM469993 | 3 | 0 | 42 | 2 | 3 | 0 | 0 |  |
| GSE11784 | GSM469994 | 3 | 0 | 19 | 2 | 1 | 0 | 0 |  |
| GSE11784 | GSM469995 | 3 | 0 | 62 | 2 | 1 | 0 | 0 |  |
| GSE11784 | GSM469996 | 3 | 0 | 23 | 2 | 1 | 0 | 0 |  |
| GSE11784 | GSM469997 | 3 | 0 | 20 | 2 | 2 | 0 | 0 |  |
| GSE11784 | GSM469998 | 3 | 0 | 46 | 2 | 1 | 0 | 0 |  |
| GSE11784 | GSM469999 | 3 | 0 | 32 | 1 | 2 | 0 | 0 |  |
| GSE11784 | GSM470000 | 2 | 0 | 48 | 1 | 4 | 51 | 1 |  |
| GSE11784 | GSM470001 | 2 | 0 | 46 | 1 | 1 | 26 | 1 |  |
| GSE11784 | GSM470002 | 2 | 0 | 27 | 1 | 2 | 9.3 | 1 |  |
| GSE11784 | GSM470003 | 2 | 0 | 24 | 1 | 4 | 14 | 1 |  |
| GSE11784 | GSM470004 | 2 | 0 | 30 | 1 | 2 | 27 | 1 |  |
| GSE11784 | GSM470005 | 2 | 0 | 38 | 1 | 1 | 15 | 1 |  |
| GSE11784 | GSM470006 | 2 | 0 | 52 | 1 | 2 | 45 | 1 |  |
| GSE11784 | GSM470007 | 2 | 0 | 41 | 1 | 2 | 18 | 1 |  |
| GSE11784 | GSM470008 | 2 | 0 | 45 | 1 | 2 | 20 | 1 |  |
| GSE11784 | GSM470009 | 2 | 0 | 53 | 1 | 2 | 16 | 1 |  |
| GSE11784 | GSM470010 | 2 | 0 | 49 | 1 | 2 | 35 | 1 |  |
| GSE11784 | GSM470011 | 2 | 0 | 38 | 1 | 2 | 19 | 1 |  |
| GSE11784 | GSM470012 | 2 | 0 | 36 | 1 | 4 | 23 | 1 |  |
| GSE11784 | GSM470013 | 2 | 0 | 43 | 1 | 2 | 22 | 1 |  |
| GSE10006 | GSM252832 | 1 | 1 | 57 | 1 | 1 | 20 | 1 | 2 |
| GSE10006 | GSM252856 | 3 | 0 | 61 | 1 | 1 | 0 | 0 |  |
| GSE10006 | GSM252857 | 3 | 0 | 37 | 2 | 2 | 0 | 0 |  |
| GSE10006 | GSM252858 | 3 | 0 | 35 | 1 | 2 | 0 | 0 |  |
| GSE10006 | GSM252859 | 3 | 0 | 38 | 1 | 2 | 0 | 0 |  |
| GSE10006 | GSM252860 | 3 | 0 | 47 | 1 | 2 | 0 | 0 |  |
| GSE10006 | GSM252861 | 3 | 0 | 36 | 1 | 1 | 0 | 0 |  |
| GSE10006 | GSM252862 | 3 | 0 | 45 | 1 | 4 | 0 | 0 |  |
| GSE10006 | GSM252863 | 3 | 0 | 45 | 1 | 1 | 0 | 0 |  |
| GSE10006 | GSM252864 | 3 | 0 | 38 | 1 | 2 | 0 | 0 |  |
| GSE10006 | GSM252865 | 3 | 0 | 49 | 2 | 1 | 0 | 0 |  |
| GSE10006 | GSM252866 | 3 | 0 | 38 | 1 | 4 | 0 | 0 |  |
| GSE10006 | GSM252868 | 2 | 0 | 37 | 2 | 2 | 0 | 1 |  |
| GSE10006 | GSM252869 | 2 | 0 | 44 | 1 | 1 | 45 | 1 |  |
| GSE10006 | GSM252870 | 2 | 0 | 48 | 1 | 1 | 15 | 1 |  |
| GSE10006 | GSM252872 | 2 | 0 | 48 | 1 | 1 | 20 | 1 |  |
| GSE10006 | GSM252873 | 2 | 0 | 50 | 1 | 1 | 38 | 1 |  |
| GSE10006 | GSM252874 | 2 | 0 | 45 | 1 | 2 | 37 | 1 |  |
| GSE10006 | GSM252875 | 2 | 0 | 46 | 2 | 2 | 23 | 1 |  |
| GSE10006 | GSM252877 | 2 | 0 | 56 | 1 | 2 | 80 | 1 |  |
| GSE10006 | GSM252883 | 2 | 0 | 55 | 2 | 1 | 70 | 1 |  |

^a^1 = COPD-smokers, 2 = CTL-smokers, 3 = CTL-nonsmokers;^b^1 = Male, 2 = Female;^C^1 = White, 2 = Black, 3 = Asian, 4 = Hispanic; ^d^packs of year; ^e^0 = early-COPD, 1 = GOLD1, 2 = GOLD2, 3 = GOLD3.
