# Supplementary material for: Gene Expression Trajectories from Normal Nonsmokers to COPD Smokers and Disease Progression Discriminant Modeling in Response to Cigarette Smoking
Source: Dis Markers. 2022 Sep 14;2022:9354286. doi: 10.1155/2022/9354286 (PMC9493146; doi:10.1155/2022/9354286)
Supplement: Supplementary 2 — Table S1: demographic data from 8 combined GEO datasets in GPL570. Table S2: demographic data from 8 single GEO datasets in GPL570. Table S3: detail demographic data from 8 GEO combined datasets. Table S4: demographic data of the validated participants. Table S5: primer sets used for real-time PCR. Table S6: predictive efficacy of single gene. [file 9354286.f2.zip › tables/Table S4.docx]

|  | | COPD-smokers | | CTL-smokers | | CTL-nonsmokers | |
| --- | --- | --- | --- | --- | --- | --- | --- |
| N | | 10 | | 10 | | 11 | |
| Age ≤60 | | 2(20) | | 5(50) | | 7(63.7) | |
| Male, % | | 8(80) | | 10(100) | | 2(18.2) | |
| Smoking, % | | 5(50) | | 10(100) | | 0 | |
| Pack years ≥20, % | | 5(50) | | 6(60) | | 0 | |
| BMI | | 22.73±2.38 | | 26.96±4.85 | | 22.80±3.40 | |
| Fan in kitchen, % | | 7(70) | | 10(100) | | 10(90.9) | |
| Good room ventilation, % | | 8(80) | | 7(70) | | 8(72.7) | |
| Often preserved food, % | | 8(80) | | 8(80) | | 9(81.8) | |
| Often cook, % | | 8(80) | | 8(80) | | 9(81.8) | |
| Comorbidity, % | |  | |  | |  | |
| Stroke | | 0 | | 0 | | 0 | |
| Hypertension | | 6(60) | | 5(50) | | 2(18.18) | |
| Diabetes | | 3(30) | | 1(10) | | 1(10) | |
| Heart diseases | | 2(20) | | 1(10) | | 1(9.1) | |
| Cough without cold | | 6(60) | | 4(40) | | 1(9.1) | |
| Phlegm without cold | | 4(40) | | 6(60) | | 3(27.3) | |
| Breathing difficulties | | 1(10) | | 1(10) | | 1(9.1) | |
| Family history of cancers | | 2(20) | | 3(30) | | 2(18.2) | |
| Family history of asthma | | 0 | | 0 | | 0 | |
| GOLD I- II | | 10(100) | | 0 | | 0 | |

**Table S4.** Demographic data of the validated participants.
