# Supplementary material for: Gene Expression Trajectories from Normal Nonsmokers to COPD Smokers and Disease Progression Discriminant Modeling in Response to Cigarette Smoking
Source: Dis Markers. 2022 Sep 14;2022:9354286. doi: 10.1155/2022/9354286 (PMC9493146; doi:10.1155/2022/9354286)
Supplement: Supplementary 2 — Table S1: demographic data from 8 combined GEO datasets in GPL570. Table S2: demographic data from 8 single GEO datasets in GPL570. Table S3: detail demographic data from 8 GEO combined datasets. Table S4: demographic data of the validated participants. Table S5: primer sets used for real-time PCR. Table S6: predictive efficacy of single gene. [file 9354286.f2.zip › tables/Table S5.docx]

| **Table S5. Primers sets used for real-time PCR.** | | |
| --- | --- | --- |
|  |  |  |
| **Gene** | **Sense primer (5′-3′)** | **Antisense primer (5′-3′)** |
| (H)NPAS3 | GAAATCTACTCTGACCAAACGC | TCTGACTTCATTGATCGTAGGG |
| (H)18S | GCAATTATTCCCCATGAACG | GGCCTCACTAAACCATCCAA |
| (H)AKR1C3 | AAGCTTTGGTCCACTTTTCATC | GGTCAACATAGTCCAATTGAGC |
| (H)MRC1 | GACGTGGCTGTGGATAAATAAC | CAGAAGACGCATGTAAAGCTAC |
| (H)CD163 | ATCAACCCTGCATCTTTAGACA | CTTGTTGTCACATGTGATCCAG |
| (H)FAM3B | CCTATGCCTACAGGTTACTCAG | TAAACTTTGTCATCGGTCCAGA |
| (H)LGALS1 | GCCAGATGGATACGAATTCAAG | GCCACACATTTGATCTTGAAGT |
| (H)AHRR | GAGACAGGAGATGATGCTATCC | TTTTCCTTGAAACTGCATCGTC |
| (H)FMO2 | GACAACTGTCCTTAGTGTGAGA | AAACCATAACTGCGTCAAAGAC |
| (H)CLEC5A | GAAAAGGATCCACATTGGCAAT | GTCGCACAGTTGAAATTCTGAT |
| (H)ITLN1 | CGGATGTAACACTGAGCACCACTG | CTCACGGCTGCTGCTGTAACC |
| (H)C3 | ACCAGCAGACCGTAACCATCCC | CAGCAGCCTTGACTTCCACTTCC |
| (H)ERP27 | CAACAGCCTCCACATGGTGACAG | GCTTGGCTGCCTTCTGGTATCTG |
| (H)KCNMB2 | TTCTCCTGGGACTGGCTATGATGG | TGATGGACGCATTCAGCAAGGTG |
| (R)Ahrr | ACTTGAGGCACCTGGACTGGAG | GGCATGTGGATGGCTCACTCTTAC |
| (R)Erp27 | ACCTGGTCTCGATGCCTAATCCTC | TCTGTGAGCCATCTGGGTTCCC |
| (R)Akr1c3 | ATGACATCGTTCTGGTTGCCTATGG | TGGAGTCCGCTTGTACTTCTTTGC |
| (R)Kcnmb2 | CTTTGAGGAGTCCATGTCCCTTGTG | ATGACGCTCTTTTGGTTCCCTTCTG |
| (R)Mrc1 | TGGACAGACGGACGAGGAGTTC | GCCACCAATCACAACAACACAGTC |
| (R)β-actin | GGACCTGACAGACTACCTCA | GTTGCCAATAGTGATGACCT |

H: human; R: rat
