# Supplementary material for: Gene Expression Trajectories from Normal Nonsmokers to COPD Smokers and Disease Progression Discriminant Modeling in Response to Cigarette Smoking
Source: Dis Markers. 2022 Sep 14;2022:9354286. doi: 10.1155/2022/9354286 (PMC9493146; doi:10.1155/2022/9354286)
Supplement: Supplementary 2 — Table S1: demographic data from 8 combined GEO datasets in GPL570. Table S2: demographic data from 8 single GEO datasets in GPL570. Table S3: detail demographic data from 8 GEO combined datasets. Table S4: demographic data of the validated participants. Table S5: primer sets used for real-time PCR. Table S6: predictive efficacy of single gene. [file 9354286.f2.zip › tables/Table S6.docx]

| **Table S6. Predictive efficacy of single gene.** |
| --- |

| genes | ROC analysis | | |  | Logistic regression | |
| --- | --- | --- | --- | --- | --- | --- |
|  | auROC | Sen. | Spe. |  | Coef. | P-value |
| COPD-smokers vs. CTL-smokers |  |  |  |  |  |  |
| FAM3B | 0.73(0.64, 0.82) | 0.50 | 0.86 |  | 1.02 | 0.000 |
| LGALS1 | 0.69(0.61, 0.78) | 0.33 | 0.91 |  | 0.75 | 0.000 |
| CD163 | 0.65(0.56, 0.74) | 0.19 | 0.92 |  | 0.49 | 0.002 |
| FAM3B + LGALS1 | 0.78(0.70, 0.85) | 0.50 | 0.87 |  | 1.01/0.76 | 0.000 |
| COPD-smokers vs. CTL-nonsmokers |  |  |  |  |  |  |
| AHRR | 0.97(0.97, 0.93) | 0.87 | 0.99 |  | 10.00 | 0.000 |
| FMO2 | 0.87(0.81, 0.93) | 0.70 | 0.82 |  | -2.33 | 0.000 |
| ITLN1 | 0.90(0.85, 0.95) | 0.81 | 0.77 |  | -1.88 | 0.000 |
| MRC1 | 0.72(0.72, 0.63) | 0.43 | 0.83 |  | 0.67 | 0.000 |
| KCNMB2 | 0.87(0.81, 0.93) | 0.65 | 0.85 |  | -2.26 | 0.000 |
| C3 | 0.90(0.84, 0.95) | 0.76 | 0.85 |  | -2.36 | 0.000 |
| AHRR+ KCNMB2 | 0.99(0.98, 1) | 96.3 | 97.4 |  | 12.3/-3.2 | 0.000/0.003 |
| CTL-smokers vs. CTL-nonsmokers |  |  |  |  |  |  |
| AKR1C3 | 0.96(0.93, 0.99) | 0.91 | 0.88 |  | 4.35 | 2.96 |
| ERP27 | 0.20(0.13, 0.26) | 0.86 | 0.63 |  | -1.4 | 0.000 |
| NPAS3 | 0.10(0.06, 0.14) | 0.88 | 0.71 |  | -3.26 | 0.000 |
| C3 | 0.16(0.10, 0.22) | 0.76 | 0.73 |  | -1.70 | 0.00 |
| AKR1C3 + ERP27 | 0.98(0.95, 1) | 0.93 | 0.92 |  | 4.8/-1.5 | 0.000 |

Sen, sensitivity; spe, specificity
